# “It’s Not Always Possible to Live Your Life Openly or Honestly in the Same Way” – Workplace Inclusion of Lesbian and Gay Humanitarian Aid Workers in Doctors Without Borders

**DOI:** 10.3389/fpsyg.2019.00320

**Published:** 2019-02-27

**Authors:** Julian M. Rengers, Liesbet Heyse, Sabine Otten, Rafael P. M. Wittek

**Affiliations:** ^1^Department of Sociology, University of Groningen, Groningen, Netherlands; ^2^Department of Social Psychology, University of Groningen, Groningen, Netherlands

**Keywords:** workplace inequality, workplace inclusion, lesbian, gay, semi-structured interviews, humanitarian aid

## Abstract

In this exploratory study, we present findings from semi-structured interviews with 11 self-identified lesbian and gay (LG) humanitarian aid workers of Doctors without Borders (MSF). We investigate their perceptions of workplace inclusion in terms of perceived satisfaction of their needs for authenticity and belonging within two organizational settings, namely office and field. Through our combined deductive and inductive approach, based on grounded theory, we find that perceptions of their colleagues’ and supervisors’ attitudes and behaviors, as well as organizational inclusiveness practices play a role in LGs’ perceived authenticity, but not belonging, in the workplace. However, these organization-level characteristics do not account for between-participant differences in perceived authenticity. Therefore, we inductively construct a typology of three groups, which we coined *conscious first-missioners*, *authentic realists*, and *idealistic activists*, based on how LG humanitarian aid workers assess and deal with not being able to be their authentic selves when they are in the field, because homosexuality is illegal in many project countries. Conscious first-missioners are separated from the other two groups based on having gone to the field once, whereby they felt in control over the decision on how to manage their sexuality. Alternatively, authentic realists and idealistic activists alike felt they did not really have a choice in how to manage their sexuality, but handled that differently. We find the importance of one’s sexuality as well as adherence to the overarching organizational mission relevant individual-level factors herein. Furthermore, we find disclosure of sexual identity to be strongly context-dependent, as participants are ‘out of the closet’ in the office, but go back into the closet when they enter the field, with different country contexts even leading to different decisions concerning self-disclosure, thus demonstrating the importance of careful sexual identity management. This so-called disclosure dilemma, we find, may not be merely an individual choice, but rather a shared dilemma involving multiple stakeholders, such as the organization and fellow team members. We discuss the findings’ contributions to existing literature on LGs’ workplace experiences and implications for future research on inclusion of sexual and other invisible minorities in the workplace.

## Introduction

Many lesbian women and gay men (LGs) across the globe work in legal and sociocultural contexts where their sexual orientation is illegal or rejected, including international LGs originating from Western countries. How does this latter group of employees, coming from an environment that is relatively ‘friendly’ toward sexual minorities, experience working in contexts where their sexual orientation can be a threat, and where they cannot be who they are? More specifically, how may this play a role in their workplace inclusion, i.e., individuals’ perceived satisfaction of their needs for belonging and authenticity in the workplace (Shore et al., 2011; Jansen et al., 2014)? Through this study, we aim to provide insights into these issues, by conducting semi-structured interviews with 11 self-identified LGs of Médecins Sans Frontières (MSF), one of the world’s leading international non-governmental organizations specialized in the provision of (emergency) humanitarian aid.

### Background

Recently, a number of countries, including most EU countries, Canada, Australia, and several US states, have adopted laws that serve to protect the workplace rights of LGs, thereby formally prohibiting employment discrimination based on sexual orientation (e.g., Colgan and McKearney, 2012; Ozeren, 2014). This development aligns with recent surveys that demonstrate generally more positive global attitudes toward Sexual and Gender Minorities (SGM; e.g., PEW Research Center, 2013; ILGA, 2016). For example, a recent large-scale worldwide survey indicated that 67% of nearly 100,000 respondents agreed that everyone should have the same human rights, regardless of sexual orientation or gender identity (ILGA, 2016). These numbers, however, do not tell the full story.

Although the situation for LGs in many Western societies has indeed improved in recent times (e.g., Colgan and Wright, 2011), the workplace remains a context in which sexual minorities run the risk of being targeted by unfair treatment, discrimination, and social exclusion (Ng and Rumens, 2017; Webster et al., 2017). This is even more so the case in many other national contexts, as LGs around the globe still face dangerous contexts because of their sexual identity. In 72 countries worldwide, homosexuality, that being sexual contact between people of the same sex, is criminalized (ILGA, 2017). Legal punishments include imprisonment, ranging from 1 month up to life sentence, and the death penalty, which is currently enforced in eight countries.

MSF offers a unique opportunity to study the dynamics of workplace inclusion of LGs working in both relatively tolerant and risky environments, for two reasons. First, international humanitarian aid staff works alternately in *office* settings in Western countries, in which Sexual and Gender Diversity (SGD) is legally protected and generally relatively accepted, and in *field* settings in project countries, in which SGD is oftentimes illegal or socially unacceptable (PEW Research Center, 2013). This creates a peculiar dynamic for LGs, since the extent to which they can disclose their sexual identity, and thus be true to themselves, is likely to be highly context-dependent. Second, the humanitarian aid sector is known for its high volatility, stress-inducing workload, and poor work-life balance (Eriksson et al., 2009; Visser et al., 2016). In such a work environment, it might be even more difficult to deal with being LG, which is an example of an invisible stigmatized identity (e.g., Ellemers and Barreto, 2006), and therefore requires careful identity management (Button, 2004).

The present exploratory study aims to contribute to the currently underdeveloped research on LGs’ workplace inclusion, by investigating humanitarian aid workers in different organizational settings (i.e., office and field). Two research questions will be answered:

(1) How do lesbian and gay (LG) humanitarian aid staff members experience that their sexual orientation plays a role in their daily work in the office and the field?
(2) Which factors play a role in LG humanitarian aid staff members’ perceptions of workplace inclusion in both office and field?

### Workplace Inclusion of LGs and the Importance of the Disclosure Dilemma

We define workplace inclusion as the individual’s perception of a specific group (e.g., the organization) providing him or her with the satisfaction of the fundamental human needs for belonging and authenticity (Jansen et al., 2014; see also Shore et al., 2011).

The need for belonging is an individual’s need to create and sustain stable relationships with others (e.g., Baumeister and Leary, 1995). Individuals fulfill this need by having recurring and positive interactions with others in a group. The need for authenticity is an individual’s need to stay true to oneself (e.g., Kernis and Goldman, 2006). This need emphasizes that group members are allowed to be different from, but also similar to other group members, as long as they are able to remain true to who they are (Jansen et al., 2014). Inclusion is different from social identification, which focuses on the processes through which the individual appreciates and connects with the group. In contrast, inclusion focuses on the signs through which the group indicates how much it wants to include the individual (Ellemers and Jetten, 2013). That is, in our definition of inclusion, the individual is the *target* and the group is the *source* of inclusion (cf. Jansen et al., 2014).

For LGs, perceptions of workplace inclusion are likely to be influenced by the disclosure dilemma (Griffith and Hebl, 2002). This encompasses a range of strategic decisions on whether, how, when, and to whom to disclose one’s invisible stigma in the workplace (Ragins and Cornwell, 2001; Clair et al., 2005; Ragins et al., 2007). A stigma consists of (one or more) characteristics that, in certain social contexts, are assessed as undesired or devalued (e.g., being LG), thus conveying a negatively evaluated social identity (Goffman, 1963; Crocker et al., 1998). Stigmas can either be visible (e.g., being overweight, being in a wheelchair) or invisible (e.g., being LG, having a mental disorder). One of the major dimensions distinguishing visible from invisible stigmas is the option to conceal the stigma. That is, whereas people carrying a visible stigma engage in *impression* management strategies, aimed at influencing others’ perceptions of the self (Goffman, 1959), people carrying an invisible stigma engage in *information* management strategies, aimed at optimally balancing potential positive (e.g., receiving social support) and negative (e.g., discrimination) outcomes of revealing the stigma (Pachankis, 2007). Indeed, disclosure of an invisible stigma is an extremely complicated phenomenon, characterized by potentially generating both benefit and harm (Chaudoir and Fisher, 2010; Spiegel et al., 2016).

For these reasons, the disclosure dilemma has been coined one of the most difficult career decisions that LGs face at the workplace (e.g., Croteau, 1996; Button, 2001). Disclosing may, under certain conditions, lead to a range of negative consequences, including ostracism, harassment, and even losing one’s job (Clair et al., 2005). Disclosure of an invisible stigma is not an ‘all-or-nothing’ phenomenon (Ragins and Cornwell, 2001; Ragins et al., 2007). Rather, it is a dynamic, continuous process of (re)negotiating of how to manage one’s invisible stigma in accordance with situational requirements (King et al., 2017), as employees may manage their stigma differently in various situations, and involving various interaction partners (Jones and King, 2014). This means that disclosure of, e.g. one’s sexual identity is highly context-dependent: whereas one might have fully disclosed in a specific context (e.g., to all close friends) and only partially in another context (e.g., only to one’s supervisor at work). Such “identity disconnects” (Ragins, 2008) have been proposed to lead to psychological incongruence, anxiety, and stress, as one is particularly vulnerable to disclosure by third parties, and thus faces continuous uncertainty with regard to who knows and who does not (Ragins, 2004, 2008).

The contextual dependence of disclosure is especially relevant for our study. LGs may face a double-edged sword with regard to self-disclosure (Chrobot-Mason et al., 2001; Griffith and Hebl, 2002): on the one hand, choosing to disclose in a particular context might lead to being discriminated against, which might lead to social exclusion. This, subsequently, might harm satisfaction of the need for belonging. On the other hand, *not* disclosing in a particular context may lead to psychological distress, due to not being able to satisfy one’s need for authenticity (Clair et al., 2005; Ragins et al., 2007). Therefore, satisfaction of one or both needs that make up inclusion might be thwarted, to the extent that a level of workplace inclusion, comparable to that of their heterosexual colleagues, may become unattainable for LGs. These dynamics may be even more pronounced in the case of international humanitarian aid work, part of which taking place in countries where SGD is illegal or socially unacceptable, and where disclosure might have potentially endangering consequences. This dependence on context may make the disclosure dilemma, with its accompanying state of anxiety and stress, even more poignant.

### Contextual Characteristics of LGs’ Workplace Inclusion

The organizational context may facilitate the disclosure dilemma for LGs, and therefore improve their workplace experiences. In line with Shore et al.’s (2011) conceptual model, we approach the organizational environment as consisting of multiple interrelated components, each providing indications to LG employees concerning their inclusion status (i.e., to what extent their needs for belonging and authenticity are satisfied), which, subsequently, shapes their perceptions of workplace inclusion.

Important components of the organizational environment include colleagues’ and supervisors’ positive attitudes and behaviors toward LGs, as well as LG-supportive organizational policies (Griffith and Hebl, 2002; Colgan et al., 2007; Ragins et al., 2007). If LGs perceive that their supervisor and co-workers treat them the same way they treat others, LGs may feel their need for authenticity increasingly satisfied. Likewise, their need for belonging might increasingly be fulfilled; such instances might lead to LG staff more positively valuing the bond with their managers and co-workers. Moreover, general human resource policies as well as LG-specific policies may positively contribute to LGs’ perceived inclusion. These include access to critical work-related information and participation in decision-making processes to all employees (Mor Barak and Cherin, 1998; Nishii, 2013), facilitation of open communication (Janssens and Zanoni, 2008), the presence of conflict resolution procedures (Roberson, 2006), and ideologies stressing the benefit of diversity (Jansen et al., 2016). For example, by facilitating open communication within the organization, interpersonal relationships between individuals might strengthen, thus improving perceptions of belonging. Similarly, active participation in decision-making processes might enhance individuals’ perceptions of authenticity, as they are asked for their contributions, based on their expertise and abilities (Nembhard and Edmondson, 2006). LG-specific policies include the establishment of a support network, sponsorship of LG-related events, or implementation of diversity trainings (e.g., Colgan et al., 2007), and are generally also expected to contribute to satisfaction of LGs’ needs for authenticity and belonging, and thus their perceived workplace inclusion (Jansen et al., 2014).

### Present Study

We assess how LG humanitarian aid workers perceive and experience their (1) *colleagues’ attitudes and behaviors*, (2) *supervisors’ attitudes and behaviors*, and (3) *organization’s inclusiveness policies* toward SGD, in which way these characteristics may shape sexual identity disclosure, and how these might relate to perceived workplace inclusion (i.e., satisfaction of the needs for belonging and authenticity). We will refer to these three elements as *organization-level characteristics*.

This study contributes to current literature in two substantive ways. First, by pinpointing the elements that contribute to or endanger LGs’ perceived authenticity and belonging, it expands existing knowledge on workplace *inclusion* of LGs, which has so far received little attention in academic literature (Ozeren, 2014; Lloren and Parini, 2016; however, see Colgan et al., 2007). Second, by explicitly focusing on employees working in contexts that substantially differ in the extent to which they have to conceal their sexuality, it enriches our knowledge on workplace *experiences* of LGs. Until now, the few studies focusing on workplace inclusion of LGs were conducted in offices (see for examples Colgan et al., 2007; Priola et al., 2014; Lloren and Parini, 2016). Particular attention is paid to how workplace inclusion of LGs differs in either office or field, where the process of disclosure of an invisible stigmatized identity carries more weight.

## Materials and Methods

### Study Setting

This study was conducted at MSF – Operational Center Amsterdam (MSF-OCA) in the Netherlands. MSF provides humanitarian assistance based on principles of neutrality and impartiality: quality medical care is provided to those who need it, regardless of race, religion or political affiliation (Médecins Sans Frontières, 2017). Examples of MSF’s activities include providing immediate basic or more specialist medical care, educating on the importance of clean water and hygienic services, auditing projects, and arranging logistics of supplies and resources within the project country.

MSF-OCA is one of five operational centers (OCs), combining MSF-Holland, MSF-United Kingdom and MSF-Germany, and housed more than 250 office employees and sent out 780 international staff members to projects in 28 countries around the world in 2016 (Médecins Sans Frontières, 2018). Humanitarian aid professionals working in a country from which they do not originate are from here on referred to as *international staff*. Examples of international staff positions are project manager, logistician, surgeon, and midwife. When employees are sent on a mission (i.e., goes into the field), they go together with other international staff members^1^. Missions on average last between 3 to 12 months, which leads to constantly changing team compositions. OCA carries responsibilities concerning the coordination of these projects, in which international staff works to provide (emergency) humanitarian aid to populations in distress, such as victims of natural or man-made disasters and victims of armed conflict, together with national staff. National staff is locally hired staff from the project country, who make up about 90% of MSF’s employees, and who can occupy positions such as nurse, doctor, engineer, and driver. In the field, international staff often lives together in international staff houses, sometimes even on guarded compounds, and tends to be separated from national staff during off-work hours.

Most employees working in one of MSF’s OCs originate from Western countries, where societal acceptance and legal rights of LGs tend to be quite well established (ILGA, 2017). To illustrate: on average, more secular and affluent countries show a tendency toward being relatively more accepting of homosexuality, with 87% of Germans, 80% of Canadians, and 60% of Americans thinking that society should accept homosexuality (PEW Research Center, 2013). Simultaneously, this study found that respondents from Africa and countries that were predominantly Muslim were least accepting of homosexuality (PEW Research Center, 2013). At least nine-in-ten respondents in several sub-Saharan countries (e.g., Nigeria), and around 85% of respondents in Pakistan and Malaysia thought that society should not accept homosexuality. As these numbers show, *on average*, international LG humanitarian aid workers’ home countries, even though there is still considerable variation, are more supportive of LGs than project countries. During the time of interviewing, MSF-OCA had ongoing projects in Nigeria, Malaysia, and Pakistan (Médecins Sans Frontières, 2017). In these, and several other countries in which participants have worked (e.g., Afghanistan, Ethiopia, Libya, Syria, Uzbekistan^2^), homosexuality is illegal and punishable. To exemplify: in Ethiopia, Malaysia, and Nigeria, same-sex individuals may receive up to life sentence in prison, and in Libya, Nigeria, Syria, and Uzbekistan, a prison sentence up to 14 years may be enforced (ILGA, 2017). Compared to an office setting, team dynamics are therefore thoroughly adapted in the field, as LG humanitarian aid workers’ decisions regarding how to handle their sexuality might have implications for themselves, their co-workers, and the organization at large, something we will discuss in detail in the results section.

Before international staff leaves for a mission, they are briefed by OCA to facilitate the transition to the field. During such briefings, MSF provides the material necessary to fulfill their job as well as possible once they are in the field. Such briefings include information on country profiles, project descriptions, the established security framework (i.e., how MSF staff is kept safe), and ways to behave appropriately in that particular country’s social setting. However, whereas these briefings provide information on different types of potentially vulnerable groups (e.g., women, certain ethnicities, and religions), so far no insights on the legal or social status of SGM-populations are provided, as became apparent throughout interviews with our participants.

### Participants

Participants in this study were 11 MSF-OCA employees, seven of whom self-identified as female and lesbian, and four of whom identified as male and gay. At the time of interviewing, five participants were married, four were in a long-term relationship with their current partner, and two were single. Of these participants, most were raised in a Western country, and almost all of them were Caucasian; the participant who was not, was of Asian origin. Age ranged from 34 to 66 (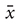 = 45.4; *SD* = 9.5). Most participants were currently mainly stationed in an office, but had experience in the field; two participants only had field experience. Jobs of participants ranged from higher organizational positions, for example during missions in the field, to intermediate positions, mostly working at OCA (e.g., in the HR or Finance department) and occasionally going on a mission.

Participants were recruited through the so-called ‘gatekeeper strategy’ (Hennink et al., 2011): Someone within the study community helped establish initial contact between researchers and participant. The Rainbow Network, MSF’s staff-run SGM support network, had a good overview of LG staff in MSF-OCA, as the result of a recent survey on SGM-inclusiveness within MSF. Therefore, their representatives were able to provide invaluable information concerning potential participants.

Given the exploratory nature of the study, the aim in participant recruitment was to find commonalities and differences across a range of participants (Hennink et al., 2011), in a first attempt to answer the research questions (also known as maximum variation sampling; Patton, 2001). A diverse range of employees was purposefully sought for, preferably differing on three dimensions: gender, nationality, and organizational tenure at MSF. Additionally, a requirement was that employees were currently not on a mission for OCA, but had been within the past 6 months; this way, we avoided that our study might have interfered directly with their activities. This also means that, during interviewing, all participants were under contract at OCA. However, not all experiences described took place during their tenure at OCA, due to the highly volatile nature of the job and relatively short contract periods at OCs. Moreover, participants were recruited both from within and outside the Rainbow Network. The participants in this study were recruited in such a way that their anonymity and confidentiality was safeguarded to the largest extent possible.

### Data Collection Procedure

Semi-structured in-depth interviews were conducted, for two main reasons. First, because of the sensitivity of the topic, building rapport between interviewer and interviewee was pivotal, and second, we were interested in participants’ individual stories and lived experiences. We considered qualitative (interview) methods appropriate in satisfying both criteria (Hennink et al., 2011). Semi-structured interviews provided the participant with the freedom to add other themes that were not part of the interview guide, thus enabling us to gain insights from our data collection and analysis inductively (Hennink et al., 2011; see also *Data Analysis* below). Furthermore, interviewees had the liberty to address topics in their own preferred order, whilst the interviewer still had the tools to guide the conversation back into a direction that allowed answering the research questions.

We derived the key questions of this study from existing literature on LGs’ workplace experiences (e.g., Colgan et al., 2007; Lloren and Parini, 2016) and the disclosure dilemma (e.g., Chaudoir and Fisher, 2010; Jones and King, 2014), as well as from literature on (antecedents of) inclusion, measured as the perceived satisfaction of the needs for belonging and authenticity (Shore et al., 2011; Jansen et al., 2014). Additionally, we added questions that we deemed relevant given the organizational setting, inquiring about e.g. safety issues and organizational support for SGM-employees in the field. Our operationalizations of the core concepts are added as Supplementary Materials.

The semi-structured interview guide was set up as follows: In the introduction, research aims and ethical issues were explained, and informed consent was obtained. Subsequently, introductory questions about the participant’s background served to collect personal information, as well as build rapport (Hennink et al., 2011). Opening questions followed, about participants’ organizational career at MSF, tenure, motivation, activities, and general work experiences at the organization, and key questions, crucial for answering the research questions (Hennink et al., 2011), ensued. These centered on contact with co-workers and supervisors, and experiences during which participants did (not) feel a member of the organization, the latter serving as a proxy to address satisfaction of the need for belonging. Questions on safety concerns ensued, followed by an in-depth focus on participants’ sexual identity. More specifically, we asked how open interviewees were in the workplace, the motivations underlying that, instances during which they felt that their sexuality (positively or negatively) affected their job and work experiences, and their involvement with the SGM-community (Rainbow Network) within MSF. These questions approximate participants’ satisfaction of their need for authenticity. Afterward, participants were invited to share their views on what the role of the organization *is*, and what it *should be*, in facilitating SGM-employees, within office and field. To conclude the interview, two closing questions were asked, in order to dissolve rapport, and end on a ‘lighter’ topic (Hennink et al., 2011). The interview guide was added as Supplementary Material, and more detailed information regarding data collection and the first author’s personal reflections are available on request.

All interviews were conducted in English or Dutch (the first author’s native language); three interviews took place face-to-face, and eight via Skype (due to geographical distance). The first author served as the only interviewer in this project. Interviews were audio recorded with participant consent, after which verbatim transcripts were made. Most information remained confidential between interviewer and interviewee; direct quotations were only published after the participant’s explicit approval. Complete anonymity of participants was guaranteed by removing all identifiable information (e.g., age, nationality, mission countries) from the transcripts; only the first author knows the participants’ identities. At the participant’s request or the first author’s judgment, counseling, provided by MSF-OCA’s psychosocial care unit (PSCU), was offered after participation; this was never deemed necessary by either party. All participants and the interviewer signed an informed consent form, which also contained the PSCU’s contact information, prior to starting each interview. This study was carried out in accordance with the recommendations of the Code of Ethics made by the American Sociological Association. The protocol was approved by the Ethics Committee of Sociology of the University of Groningen. All subjects gave written informed consent in accordance with the Declaration of Helsinki.

### Data Analysis

Grounded theory (Corbin and Strauss, 2008) guided data analysis, combining an inductive and a deductive approach (Hennink et al., 2011). Based on our conceptual model, the first author developed a deductive codebook, containing 41 codes, which was then discussed extensively with the second author. The subsequent analytical process can be described as a predominantly iterative, cyclical process of theoretical reflection, data collection and data analysis, which means that we were open to inductive insights gained during data collection and analysis (Hennink et al., 2011). Data were coded inductively through descriptive coding (Saldaña, 2009), meaning that the main topic of a certain fragment of the interview was summarized in one word. The first author developed 52 inductive codes, identifying new themes and topics emerging from the data, leading to a final codebook containing 93 codes. The second author then independently crosschecked a portion (*n* = 4) of the interviews using that codebook, to examine consistency in coding across interviews, and to guarantee some form of interrater reliability. Afterward, first and second author discussed the coded interviews, based on which coding was refined. The transcripts were analyzed with the help of the qualitative data analysis software program Atlas.ti 7 (Friese, 2013). The codebook is available as Supplementary Material.

Data analysis continued by placing the deductive and inductive codes that shared a common attribute into the same overarching categories (i.e., categorization, Hennink et al., 2011, p. 246) which allowed us to obtain a better conceptual understanding of our data (see Hennink et al., 2011). More specifically, we created several overarching categories consisting of multiple subcategories. An example is the category ‘inclusiveness practices.’ Subcategories herein are ‘diversity,’ ‘equality,’ ‘initiatives,’ ‘policy,’ and ‘organizational support.’ These categories and subcategories are provided as Supplementary Materials. This categorization allowed us to create multiple groups based on the way participants spoke of their perceived authenticity, and it helped establish the underlying phenomena shaping these perceptions (i.e., comparison; see Hennink et al., 2011, p. 243). Additionally, we constructed so-called thick descriptions (Hennink et al., 2011, p. 238) of our main variables of interest (i.e., authenticity and belonging), providing the multiple dimensions of which participants spoke in addressing these variables. Examples for authenticity include ‘being able to share personal stories,’ and ‘seeing it as their human right to be open.’ Examples for belonging include ‘always a member,’ and ‘all together.’ These thick descriptions are added as Supplementary Materials.

After categorization and comparison, conceptualization proceeded with exploring the expected links between the individual elements within the data (Hennink et al., 2011, p. 247) by relating the findings to the deductively developed conceptual model. This process was mainly based on two strategies: looking for the ‘big picture’ in the data, and taking a step back to gain a broader overview of the issues, whilst simultaneously moving closer to the data, in order to examine certain details within the data, especially by comparing differences between individuals. An example is the analysis of how open participants were about their sexual identity in the field. In examining this, we looked at how this related to perceptions of workplace inclusion, and how it was embedded in existing organizational arrangements, thereby accounting for the ‘big picture.’ Simultaneously we looked for the specific details explained by our participants with regard to the reason(s) for how open they were, paying attention to differences between individuals (Hennink et al., 2011).

During the process of data collection and -analysis, the authors worked in accordance with the criteria necessary to ensure trustworthiness of a qualitative study, such as transferability (to assess to what extent findings are generalizable) and confirmability (to assess to what extent findings are based on the data, not the researcher’s predispositions) (see Guba, 1981 in Shenton, 2004). More detailed information regarding the data analysis process is available on request.

## Results

Below, we first discuss participants’ perceptions of *belonging*, and highlight aspects that create minor differences in the degree to which participants feel belonging to the organization. As we found little difference in participants’ fulfillment of the need for belonging, we devote relatively little attention to this topic. Second, we describe participants’ perceptions of *authenticity* in detail, since we found noticeable differences between office and field, as well as between participants. Third, we illustrate the inductively emerged *individual-level* factors related to variation in perceived authenticity between participants, as this variation was only *partially* related to the three *organization-level* characteristics we distinguish.

### Description of the Belonging Dimension

Participants, almost unanimously, felt clearly that they belonged to MSF. As illustrated by one participant, who felt that “you’re like the firefighter […] you’re called on a Saturday, you’re called on a Sunday, you’re called […] at 1AM in bed. So, I think there’s never a moment that I do not feel part of it.” (M^3^, 7½ years’ experience in office and field). Another participant expressed that “MSF has very much a sort of family feel to it” (F, 3 years’ experience in office and field).

MSF was lauded for its effectiveness in creating strong feelings of belonging, and thus of showing signs of employees’ inclusion, for example through organizational artifacts such as t-shirts, training and welcome days, and the yearly general assembly meetings. These high levels of belonging were partially due to the inherent nature and work of the organization, as participants felt even more strongly belonging to the organization any time they were contributing to fulfilling MSF’s organizational mission (‘to preserve life, restore dignity, alleviate suffering, and protect people’s ability to make their own choices’).

Nonetheless, two factors slightly hampered perceptions of belonging. The first was a lack of organizational tenure, which subsequently led to not having many social relationships within the organization. Within MSF, going into the field is a way of gaining status among peers, a notion voiced by multiple participants. As such, several of them recalled that, when they had just joined the organization, they still had to ‘prove’ themselves. As one participant explained, “MSF does tend to be quite suspicious of outsiders […] so I think that’s […] the flip side of being a very close-knit community; it doesn’t always want to let people in if you’re not part of the club” (F, 3 years’ experience in office and field).

Another factor decreasing belonging was rooted in the inability to share personal stories with co-workers, mainly in the field, where some participants felt they could not be completely open about themselves, especially about their sexual identity. One participant said, “I think primarily […] it can be lonely. As everybody’s talking about their family, and […] I end up talking about my brothers and sisters instead, you know?” (F, 6 years’ experience in the field).

In sum, all participants expressed strong feelings of belonging to MSF, primarily resulting from the nature of the organization and its humanitarian aid work. Factors hampering participants’ perceived belonging were shorter organizational tenure and an inability to share personal stories due to one’s (sexual) identity.

### Description of the Authenticity Dimension

Whereas interviewees generally shared a strong sense of belonging, this was not the case for their perceived authenticity. This partly applied to their experiences in the office, but mostly to those in the field, where participants felt they could not always be themselves. Below, we first discuss perceptions of authenticity in the *office*, followed by a discussion of authenticity perceptions in the *field*. Herein, we recognize that the analytical distinction between office and field may be less clear-cut in real life, where support received by office co-workers may mitigate the negative consequences of being closeted in the field.

#### Authenticity in the Office

In the Amsterdam office, participants generally felt enabled and encouraged to be authentic, by colleagues and supervisors alike, and it was perceived as a pleasant work environment where participants could be themselves. Therefore, all participants were open about their sexual identity in the office. As an example, several participants recalled their colleagues’, mostly neutral to positive, responses to the disclosure of their sexuality within the Amsterdam office, which they described as largely characterized by genuine interest in their family life. Participants mainly ascribed this to the general atmosphere in Amsterdam, and the Netherlands at large, which, compared to other countries, was seen as tolerant and open-minded toward sexual minorities. They did not seem very concerned about the disclosure dilemma: multiple participants mentioned not having had severe difficulties coming out to their colleagues and supervisors within the office.

They emphasized the diverse nature of the organization, especially in terms of cultural background of employees, which created the idea that participants could be open about their sexuality. One participant said: “I felt it was a very comfortable environment […] to be out, which was great, and that was, you know, also a part of what made it feel very comfortable and a good experience to join the organization” (F, 3 years’ experience in office and field).

Especially the Rainbow Network, established in 2016 in order to promote SGM-inclusivity within Amsterdam HQ and the other OCs, was perceived to be a safe haven by several participants. One participant sketched it as a place

“… to support each other in those moments when […] you do not feel part of the organization. And *all of us* know those moments, and it’s just really nice to be able to talk about it, and that there’s people who just *get it* [emphasis added]” (F, 3½ years’ experience in office and field).

Despite the above, participants also voiced concerns about the boundaries of that generally supportive work environment. Some of them had heard negative comments about their own or others’ sexual orientation: jokes, negative remarks, and sexual innuendos were made in the office (so-called microaggressions; cf. Sue, 2010). Some participants mentioned colleagues’ efforts to set them up with ‘the other gay person in the office,’ simply because they were both homosexual. Another participant shared an example, of having to remarry her partner in different countries, given that their marriage in the Netherlands was not legally binding everywhere. Sharing this with her supervisor evoked the remark that “they must really love getting married.” These examples illustrated a lack of awareness of some of the particular situations faced by SGM-workers.

A Rainbow Network questionnaire administered in 2016 further exemplified this paradoxical situation. In this questionnaire, MSF employees, both cisgender^4^ and heterosexual, as well as SGM, were asked about experiences with homophobic, transphobic, and heterosexist comments within MSF. The questionnaire accumulated responses from almost 300 employees, of whom 62% witnessed derogatory language or inappropriate jokes when no openly SGM-individuals were present. Moreover, 35% of respondents had been present while such comments were made directly to SGM-employees^5^ (M. Schoonheim, personal communication, September 30, 2016).

Several participants stated that MSF, as an organization, was still ‘in the closet,’ since issues concerning sexual orientation of employees have only recently started receiving attention. Participants ascribed this to the organization’s very clear organizational mandate to deliver the highest quality humanitarian aid to those who are most in need, making everything not directly related to fulfilling this mandate of secondary importance. Another frequently mentioned argument was that some colleagues and supervisors believed that, given that they were situated in Amsterdam – one of the most gay-friendly capitals in the world - acceptance of sexual minorities had been fully achieved and did not need further improvement.

Taken together, participants’ stories sketched a paradoxical situation within the Amsterdam office. Although they felt encouraged and enabled to be themselves, and believed the office generally provided a pleasant work environment, there were boundaries to the tolerance they experienced (cf. Buijs et al., 2011). This came to the fore in the form of jokes, derogatory remarks, and other microaggressions (Sue, 2010), a certain hesitation to discuss sexual orientation issues within the organization, and an experienced lack of organizational policies directed at SGM-issues. All these aspects combined gave participants the idea that there still was ample ground to gain within OCA.

#### Authenticity in the Field

In general, participants felt their need for authenticity far less fulfilled in the field, compared to the office. Participants all went ‘back into the closet’ when going on a mission. This decision was strongly rooted in perceived risks of being LG in many project countries. Such risks included the potential of disturbing team dynamics and the possibility of being outed by someone (i.e., when one’s sexual orientation is involuntarily revealed to a third party). Due to these risks, participants needed to gauge their fellow international staff members on their viewpoints toward SGD, which was difficult, as there was a large degree of uncertainty. That is, participants did not know how their fellow international staff would react to their coming out. Several participants spoke of going into the field assuming that their teammates would be able to handle such information discretely, which they oftentimes did not find to be the case. Interestingly, they mainly ascribed this to the organization not clearly communicating what was expected of international staff members, as international staff members were not instructed about the boundaries of acceptable behavior among colleagues.

Participants thus had to decide very carefully whether, and, if so, to what extent, how, and to whom to disclose their sexual orientation within the international staff team. Here, it deserves mentioning that, throughout the interviews, participants did not speak of the disclosure dilemma concerning national staff or beneficiaries: None of the participants had come out to national staff members or beneficiaries, and no one was planning to do so in the future. Given this unanimity in our sample, we will therefore not discuss this aspect in further detail. The magnitude of the disclosure dilemma was therefore considerable, as illustrated by one of our participants, who said, “you’re living together, working together, and you’re totally reliant on each other. The team dynamic is everything. And you have something that alters the team dynamic” (F, 7 years’ experience in office and field). This uncertainty with regard to sharing information about their sexuality within the international team was related to LGs’ fear of being outed to national staff, which might have negative consequences in legal or social contexts where homosexuality is illegal.

These negative consequences of being outed can range from national staff no longer wanting to work together, to beneficiaries (i.e., the people receiving the aid provided by MSF) deciding they no longer want to be treated, since the organization may be perceived to support something as sinful and unnatural as homosexuality. If that were to happen, MSF might lose its entire “raison d’être” since providing support for beneficiaries is the organization’s primary concern. More severely, being out or outed might affect MSF’s position within the project country; its acceptance in local communities is paramount to doing their job, as one participant expressed: “We have to be able to retain our local operating position. And if the local moms or the local bishops decide that MSF is pursuing a queer rights agenda, it may become virtually impossible to safely stay there” (M, 12 years’ experience in the field). Participants expressed a thorough understanding of the organization’s difficult position in this respect.

Depending on the context, being outed may even lead to being taken out of the mission, to guarantee the individual’s and their team members’ safety (as they might be tried according to local laws). Given these strong associated risks, a pivotal role in LGs’ dealing with identity management issues in the field could be played by their supervisor(s) in the field^6^. Currently, supervisors did not deal particularly well with participants’ self-disclosure, as illustrated by one participant:

“I told my project coordinator I had a wife, and he was European, he was like ‘I don’t care.’ And I’m like ‘I don’t think you do, as a person. But as a manager, do you know how to manage the situation? Like, if something came up, and there was an issue within the team, would you know how to manage that within the context of the field situation?’ […] I don’t think *he’s* being trained on how to manage it (F, 6 years’ experience in office and field).”

Given the perceived risks and potential consequences of being open about being LG, participants generally considered it better to go completely back into the closet. Interestingly, MSF regarded going back into the closet as the *only* feasible way to manage one’s sexual identity in the field, as mentioned by several participants. That is, the organization’s advice on handling being LG in the field was to ‘don’t ask, don’t tell.’

Being back in the closet, however, was not always easy to sustain for participants. Considering they were able to be open about their sexuality within the Amsterdam office and their private lives, participants found it challenging to live a closeted life in the field, as one participant illustrated:

*“Wherever* you go, in *any* project, *wherever* in the world, the first thing people ask is: ‘Are you married?’ Because in most cultures where we work, your marital status and your parental status, so having children, are the things over which you bond” (F, 3½ years’ experience in office and field).

Many participants struggled with how to maintain this straight façade, especially during their first mission. Some of them recalled having had to make an instant decision as to how they were going to approach questions concerning their family life. An influential factor was the duration of the mission. As was mentioned by multiple participants, it is easier to keep up the façade if one is in the field for a shorter period (e.g., 2 weeks) than when one is away for longer (e.g., 6 months). Another factor was participants’ relationship status. Understandably, participants in a relationship experienced more difficulties regarding questions about their family life than single participants. For example, when they had a Skype conversation with their partner, they had to pretend their significant other to be a mere friend. Other participants who had children found it hard to deny their existence, as revealing this might have evoked further inquiries about their family life.

In sum, participants mentioned many difficulties to fulfill their need for authenticity, with regard to their sexuality, given local laws and social norms in project countries. In the majority of cases, they thoroughly compromised their authenticity, and they felt that MSF did not provide sufficient support to combine doing their job well with simultaneously managing their sexual identity. If the specific context made disclosure a highly risky endeavor and participants felt that disclosing could sincerely harm the team, the project, and the organization, they went back into the closet. Furthermore, participants sometimes did not trust their teammates and supervisors to deal well with potential disclosure, further strengthening the perceived necessity to live a closeted life in the field. Hence, contrary to what much literature assumes, we found that our participants spoke of disclosure as more strongly related to the potential risks within the particular *organizational* context, and less to negative *personal* consequences, such as running the risk of discrimination or social exclusion (e.g., Ng and Rumens, 2017; Webster et al., 2017).

### Differences in Perceived Authenticity Between Participants

Next to the general patterns described above, we discovered variation in how participants responded to compromised authenticity. That is, although all participants mentioned lower satisfaction of their need for authenticity in the field, we found differences *between participants* in how they handled this. Differences were mostly rooted in the importance they attached to their sexual identity, combined with the salience of the organizational mission. Based on these differences, we inductively generated three groups of participants, which we labeled *conscious first missioners*, *authentic realists*, and *idealistic activists* (see Table 1 and explanation below).

**Table 1:** Characteristics of participants, participants’ expressed belonging and authenticity, and group categorization.

Group	Defining individual characteristic	ID	Gender	Tenure	Belonging	Authenticity
*Conscious first missioners*	Degree of autonomy in	03	F	1 year	Low due to being new	Satisfactory
	deliberately and consciously	07	F	2 years	Very high, but low when new	Satisfactory
	managing the disclosure	10	F	3 years	High, but low when new	Comfortable
	dilemma					
*Authentic realists*	Strong adherence to the	04	M	7½ years	Very high	Satisfactory
	organizational mission paired	05	F	11½ years	High	Satisfactory
	with lower sexual identity	08	M	12 years	Very high	Satisfactory
	centrality to the self	11	M	6½ years	High	Comfortable
*Idealistic activists*	Contextual dependency of	01	M	12 years	Low due to poor support in field	Unsatisfactory
	self-disclosure in the field	02	F	3½ years	High	Unsatisfactory
	paired with relative importance	06	F	7 years	High	Mediocre
	of sexuality to the self	09	F	6 years	Low due to loneliness in field	Mediocre
Average				6½ years		


As already inherent in the label *conscious first missioners* (a commonly used term for MSF staff who go into the field for the first time), participants categorized in this group (*n* = 3) had, when the interview was taken, only been in the field once. They expressed the perceived choice they had with regard to self-disclosure. That is, they made a deliberate decision concerning the (non-)disclosure of their sexuality to others while they were in the field, which was less pronounced in interviews with the other participants.

The other eight participants, whose experience in the field ranged from having been on several missions to having over 100 months of field experience, showed substantial differences in perceived authenticity. We derived two more groups based on how these participants described *the relationship between their sexual orientation and the organization’s mission*. Certain participants voiced an understanding to put their sexuality ‘on hold’ while they were in the field, whereas others saw it as their fundamental human right to be who they were, also in the field. We named the second group the *authentic realists* (*n* = 4), because they were accepting of the necessity to hide their sexual identity, whilst their perceived authenticity was relatively unblemished. Finally, we labeled the third group the *idealistic activists* (*n* = 4), because in their stories they delved into a more idealistic perspective of handling sexual orientation issues at MSF, and because they all actively advocated toward that aim.

#### Conscious First Missioners

Insights described by the conscious first missioners (*n* = 3) were especially relevant regarding their field experiences. They were relatively critical toward the organization for its lack of support of sexual minority employees. This may be the case because, compared to other participants, they may, due to their short tenure at MSF, have been more prone to mirror the sexual orientation policies of their previous employer with those of MSF.

The conscious first missioners can especially be set apart from the other two groups because they had only been in the field once, and they made a *deliberate* and *conscious* decision with regard to their (non-)disclosure. That is, whether they decided to go back into the closet or disclose their sexual orientation, they described feeling in control over that process, as they themselves were responsible. One participant phrased this as follows:

“I just didn’t want to kind of create a problem there. So I did kind of get back in the closet, I suppose, for that period of time. Which was a conscious decision that I felt I was happy with, because it wasn’t so important for me to be out, if it was going to have a detrimental effect on […] the team or the project work that we were doing (F, 3 years’ experience in office and field).”

Making a conscious decision regarding (non-)disclosure might mean there was a sense of autonomy present among the conscious first missioners. Psychological theories of needs describe the importance of a sense of autonomy, as it is related to freedom, and thereby to positive individual functioning (e.g., Deci and Ryan, 2000). Having had the perception that they themselves were responsible for choosing to what extent they were being authentic to their sexual identity, conscious first missioners might have felt that – irrespective of having disclosed their sexual identity or not – their need for authenticity was not seriously compromised. This stood in stark contrast with how other participants, i.e., the idealistic activists, described the (non-)disclosure process: they more often felt forced by the organization to go back into the closet, thereby taking away that feeling of autonomy.

#### Authentic Realists

The authentic realists (*n* = 4) encountered relatively few problems with their sexuality. In general, they were less bothered by nasty comments than the other participants were. An explanation for this might be found in what has been termed identity centrality or -importance (Settles, 2004). This refers to the idea that people fulfill multiple roles and are members of multiple groups, which together serve as sources of their identity. Each of an individual’s identities may be of higher or lower importance to the individual. Indeed, all participants categorized into this group emphasized that they considered their sexuality as central to their self-concept to a limited extent only. The authentic realists downplayed the importance of their sexuality in the workplace, and instead emphasized their salient identity of a humanitarian aid worker. This resonates with theorizing along the lines of self-categorization theory (SCT; e.g., Turner et al., 1987), which proposes that a certain identity can become more or less salient, depending on the context.

In the field context, it was obvious that the authentic realists considered their sexual orientation as not important, meaning that this part of their identity was not salient at all when at work. Instead, their identity as a humanitarian aid worker was extremely salient within this context. This was strongly linked to their support for MSF’s organizational mandate, to which they all referred. MSF, they asserted, should not be concerned with their employees’ sexuality, as it did not directly contribute to fulfilling its mission. They did believe the organization held a certain duty of care to its SGM-employees, but, according to them, this should not be given priority on the organizational agenda. Instead, they believed that MSF has “bigger fish to catch,” for example by providing aid to SGM national staff or beneficiaries.

The authentic realists were acutely aware of why they went into the field in the first place, which was to help those in need. They claimed they already knew this before they joined the organization, which made going back into the closet easier. Similar to the conscious first missioners, they did so voluntarily, as that was needed in order to work for MSF, and to do their job well. However, in contrast with conscious first missioners, authentic realists never spoke of any element of choice; they felt going back into the closet was the only viable way to deal with their sexuality in the field. One participant illustrated this, arguing that

“This is a very fulfilling rewarding and challenging role, that I am proud to do, and that I want to do well. And part of that does simply mean saying ‘Okay! I am not a sexual person right now. I am not a personal person right now!’ And I don’t mind that! I mean, I do think it’s not necessarily psychologically easy. But I have found a balance that works well for me” (M, 12 years’ experience in the field).

In sum, despite not being able to be one’s authentic self, the participants categorized into this group were relatively accepting of the situation. They referred to the organizational mandate, and ascertained that issues surrounding sexual orientation were relatively unimportant within MSF. Moreover, they knew that, in order to work in the field, they had to conceal their sexuality, which they were willing to do.

#### Idealistic Activists

The idealistic activists (*n* = 4) were, comparatively, more critical with regard to sexual minority issues within MSF. They argued that MSF should be more explicit about the differential treatment of any minority group (not just sexual minorities), about which the organization was not very vocal. The office was the most suitable context for change, according to these participants. All four participants categorized into this group were actively contributing to such change, as they were advocating for more inclusivity toward minority groups within MSF.

They were frustrated about the lack of attention for sexual orientation issues within the organization. As an example of the absence of organizational policies tailored to protect the lives of sexual minority employees, participants repeatedly made a comparison with the extensive preparation and security briefings employees received before they went into the field. These briefings contained information on gender, ethnicity, nationality, and religion, for example in terms of the social norms and practices in a particular country, and how minorities might be regarded there. However, there was no specific attention to sexual orientation issues, which they found surprising and frustrating, given the often very negative way that SGM are perceived in different field contexts.

When idealistic activists went into the field, they were comparatively less willing simply to conceal their sexuality, and to accept that they cannot show their true sexual identity solely because this would not be possible in the given legal or social context. In their explanations underlying this, they ascertained that their sexuality is inextricably part of who they are, and that they saw it as their human right to be open about who they were. As one of them said:

“Some people, they closet themselves, they do their work, and they just ignore it. And that’s certainly one way of coping, but it shouldn’t have to be the only way to cope. You should have a choice in how authentic you are to yourself. Within safe parameters, with an employer who is aware and who can guide on how that might look in the field” (F, 7 years’ experience in office and field).

With regard to having a choice in how authentic one is, two participants spoke of being outed in the field. This made them feel vulnerable, led to a breach of trust within the team, and required them to become even more vigilant. Such occurrences can also be linked to a deprived sense of autonomy (introduced above), because the element of choice in disclosing one’s sexuality is removed, and may therefore contribute to a diminished satisfaction of one’s authenticity need.

Participants in this group assumed that the organization currently had a particular mindset concerning how sexuality issues should be dealt with, namely of not wanting to impose “Westernized” norms and values, such as homosexuality, onto project countries. Therefore, the organization saw only one feasible way of dealing with homosexuality, which was to strongly urge LG staff to go back into the closet in the field. In these participants’ opinions, the organization was hereby giving the signal that they did not want to delve into sexual orientation issues, simply because it might be a minefield.

Interestingly, all participants categorized into this group, as well as two of the conscious first missioners, mentioned the need to differentiate between contexts; that is, they believed disclosure should not always be considered an immediate threat to staff safety and organizational legitimacy. Instead, they urged the organization to consider contextual differences with regard to the extent to which disclosure of an LG identity should be possible, instead of the general ‘don’t ask, don’t tell’ advice currently given. This illustrated participants’ recognition that disclosure is not always and everywhere possible, but that the extent to which it *is* possible was strongly dependent on the particular country context.

Idealistic activists believed that sexual orientation issues should be discussed within MSF, because it is a lived reality for a part of MSF’s employees. One participant described this sentiment as follows:

“I feel like they have their blinkers on when it comes to this, and I feel like it is always in the ‘too hard basket.’ Which is ironic, because MSF has never accepted a mission to be in the ‘too hard basket.’ They are *without borders* [emphasis added]! Don’t tell them they can’t go there, because they’re going to go! And I think: ‘why wouldn’t you want to go here?”’ (F, 7 years’ experience in office and field).

In sum, idealistic activists were concerned with the organization not taking any stance on sexual orientation issues, which they found frustrating and disappointing. In making this claim, they referred to the organizational boundary of care, which should also include taking care of *all* employees, as well as to their fundamental human right to be who they were. They understood that the organization was in a difficult position in this respect, but believed that nevertheless changes could and should be made.

Taken together, in this section, we saw how participants spoke in different ways of how important their sexual identity was to them, and how much they perceived to be able to be authentic in the field. Participants’ stories highlight the importance of *individual-level* differences in sexual identity management when trying to understand differences in perceived authenticity within the workplace. Through this inductively constructed typology, we received further insights into what could potentially contribute to variations in perceived authenticity.

## Discussion

### Contributions

This study had two aims. First, it mapped LG humanitarian aid workers’ experience how their sexual orientation plays a role in their daily work, both in an office and a field setting. Second, it identified organizational factors that may play a role in the perceptions of workplace inclusion among this vulnerable minority group. As a result, this study makes four main contributions.

First, our findings *corroborate* previous research on sexual minorities’ workplace experiences. Participants experienced themselves, or witnessed someone else, being targeted by jokes, derogatory comments, or other types of micro-aggressions (Sue, 2010). This is in line with the well-established finding that SGM-employees still face subtle and not-so-subtle forms of discrimination in the workplace (e.g., Griffith and Hebl, 2002; Ozeren, 2014; McFadden, 2015). Accordingly, they treat the disclosure dilemma with caution, especially when in the field (e.g., Ragins and Cornwell, 2001; Griffith and Hebl, 2002; Clair et al., 2005; Ragins et al., 2007).

Second, we *extend* current research on workplace experiences of sexual minority employees by comparing an office to a field setting, thereby revealing an additional range of issues faced by this vulnerable group. Firstly, our data reveal that disclosure of sexual identity is highly dependent on the context of work. Whereas all participants chose to be open about their sexuality in the office, the opposite was true for the field. There, participants unanimously went back into the closet. Nevertheless, even within different field settings, participants mentioned different degrees to which they considered disclosure as possible, thereby further illustrating its high context-dependency. Secondly, team dynamics may be vital to the outcome of a mission. As disrupting these by disclosing one’s sexuality might lead to a breach of trust within the team, the importance of the disclosure dilemma increased considerably in the field. Thirdly, our findings also shed light on how LG workers manage their sexual identity to people outside of the organization. When they were in the field, deciding strategically how they would portray their sexuality (cf. Orne, 2011), became even more vital than in the office. This may not only affect the individual, but also their team and the organization at large, for example by affecting safety or organizational legitimacy. This issue is particularly prominent given the importance of family as a discussion topic within most of MSF’s project countries.

Third, our findings also suggest extending the notion of an individual disclosure dilemma to that of a *shared* disclosure dilemma. Under specific conditions, the organization (here MSF) may have an interest in *not* enabling SGM-employees to disclose, thereby thwarting their opportunity to satisfy their need for authenticity. This is most likely in situations where disclosure might endanger the safety of specific LG employees, but also for MSF as a whole. Similarly, fellow international staff members in the field may also be stakeholders in this dilemma. For example, team dynamics may change once someone reveals their sexuality within the team, with potentially detrimental effects on fellow staff’s ability to do their job well. Whereas the disclosure dilemma is usually presented as an *individual* dilemma (e.g., Griffith and Hebl, 2002), we found that multiple parties may be involved in and affected by this dilemma.

Fourth, participants experienced workplace inclusion differently depending on the focal organizational setting. We encountered considerable variation in the extent to which their need for *authenticity* was satisfied in either office or field. Simultaneously, we found *little* variation in the extent to which their need for *belonging* was satisfied, as this was almost constantly fulfilled, in both office and field. Satisfaction of one’s need for belonging was only slightly hampered by limited organizational tenure. Similarly, we found an inability to talk about one’s personal life in the field to also inhibit complete satisfaction of the need for belonging. This, in turn, strained satisfying one’s need for authenticity and perceived workplace inclusion.

It has been hypothesized that human beings seek to optimize satisfaction of both needs for belonging and authenticity, leading to “optimal” inclusion (Shore et al., 2011; Jansen et al., 2014) or optimal distinctiveness (e.g., Brewer, 1991) within their social groups. Our findings suggest the possibility that if one of these two fundamental human needs is largely satisfied, there might be a sort of trade-off, whereby lower satisfaction of the other need may be compensated. In our study, decreased satisfaction of the need for authenticity was relatively acceptable for our participants, given that they received a surplus on the satisfaction of their need for belonging. This finding may point to a gap in the current definition of inclusion: by focusing on a minority group possessing a *concealable* characteristic, we can see the relative importance of fulfilling the need for authenticity, compared to the need for belonging. This raises new questions: is the construct of inclusion currently well defined, in its assumption that both needs for authenticity and belonging are equally important, or may the relative importance of one need trump the other under certain circumstances?

### Implications

The second aim of our paper was to deduce the organizational factors that may play a role in the perceptions of workplace inclusion among LG humanitarian aid workers. We expected *colleagues’*, *supervisors’ attitudes and behaviors*, *organizational inclusiveness practices* and the disclosure dilemma to play a role in perceptions of inclusion (see Shore et al., 2011). We suggest two main implications: First, the abovementioned *organization*-level characteristics can, to a certain extent, account for variations in perceived workplace inclusion of LG humanitarian aid workers. However, second, in order to account for *between-participant* differences in perceived authenticity, we propose to also consider *individual-level* characteristics.

Firstly, we found colleagues’ attitudes and behaviors to be strongly related to perceptions of workplace inclusion among LG humanitarian aid workers. All participants felt welcomed and encouraged to be authentic by their co-workers within the office. Participants valued being able to share their experiences and to be open about their sexuality. This was pivotal, given the considerable contrast with the field, where, attitudes and behaviors of national staff and beneficiaries were far more negative. Therefore, participants were especially happy to witness the openness in the office. However, openness toward SGM-employees also showed a Janus face (cf. Cramwinckel et al., 2018). Even though co-workers generally reacted neutrally to positively when participants spoke of their sexual identity, and were supportive and understanding of their position, participants still faced subtle forms of discrimination, for example through being targeted by jokes and other micro-aggressions (Sue, 2010).

Secondly, supervisors’ attitudes and behaviors also affected perceived workplace inclusion. Within the office, these were generally considered neutral or positive; in the field, the mission understandably strained inclusion perceptions. Almost all participants therefore raised concerns about MSF’s training programs, as the organization is currently not equipping neither employees nor supervisors with the appropriate tools to deal with the presence of sexual minority staff in the field. Participants suggested that trainings could be offered, to raise awareness and understanding of sexual orientation issues (cf. Griffith and Hebl, 2002).

Thirdly, participants assessed MSF’s current organizational inclusiveness practices toward sexual minorities to be non-existent. Despite the organization’s extensive and careful efforts to establish an impeccable security framework, sexual minority issues are currently not covered. Including sexual minorities in this framework, as is the case for women and people of certain religions and nationalities, could be a first step toward more SGM-inclusiveness, by ensuring equal treatment to other vulnerable minority groups. Alternatively, extending the perspective of the authentic realists, we could see another role for the organization: MSF could also offer support by focusing on the vulnerable group itself, e.g., by teaching SGM-employees coping strategies on how to deal with lower fulfillment of their authenticity need when they are in the field.

At least within the office, it seems that these three organization-level characteristics could somewhat facilitate the disclosure dilemma for LG humanitarian aid workers. In the field, however, it is an entirely different story, because the organization cannot do much, and has to maneuver within specific legal and cultural frameworks. Participants understood this issue very well. Most participants therefore saw challenges for the organization, for example in finding feasible ways, given these constraints, to facilitate the disclosure dilemma, and thereby contributing to increased workplace inclusion of sexual minority employees.

Research may benefit from including more fine-grained individual-level factors in studying workplace inclusion. The *interplay* between individual-level and organization-level elements may provide an especially fruitful focus (cf. Clair et al., 2005; Wax et al., 2018). Several *individual*-level characteristics turned out to be more strongly associated with perceived authenticity and workplace inclusion than *organization*-level characteristics. In this, we distinguish individual-level characteristics that accounted for variation in perceived authenticity. Firstly, as ‘conscious first missioners’ experienced control over whether to disclose their sexuality when in the field, they did not perceive their need for authenticity as heavily compromised. These participants may have perceived a *degree of autonomy in the disclosure dilemma*, which was absent for participants categorized into the other two groups. Secondly, although ‘authentic realists’ did not experience autonomy in the disclosure dilemma, they did not see their need for authenticity as heavily compromised, which was related to two components. They demonstrated a *strong adherence to the organizational mission*, as well as voiced that their *sexual identity was a less central part of their identity*. Thirdly, ‘idealistic activists’ also did not experience autonomy in the disclosure dilemma, but they *did* perceive their need for authenticity as compromised. We related this to two other individual-level components, namely the belief that sexual orientation issues have to be discussed within the organization, because of the strong *contextual dependency of self-disclosure in the field*, and the *relative importance of their sexual identity to the self.*

**FIGURE 1:**
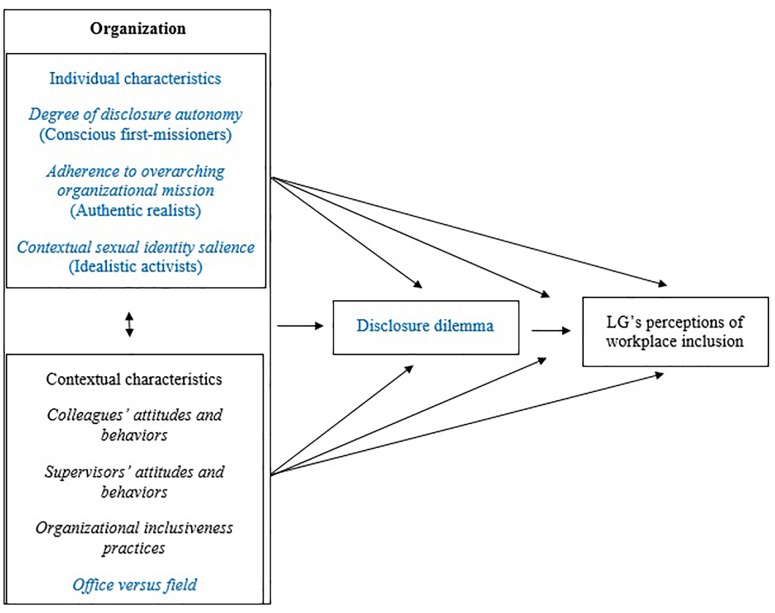
Updated conceptual model, integrating heavily simplified elements from the Shore et al. (2011) model with the findings presented in our manuscript. Specifically, the elements we add to represent the particular context of our case study (represented in blue), are: (1) The individual-level characteristics which we found to play a role in lesbian and gay humanitarian aid workers’ perceptions of workplace inclusion: (a) Degree of autonomy (i.e., perceived control over the decision to disclose or not, for conscious first-missioners). (b) Adherence to the overarching organizational mission (i.e., the extent to which the LG aid worker is willing to put the organizational mission before everything else, for authentic realists). (c) Contextual sexual identity salience (i.e., the extent to which the LG aid worker finds their sexuality to be a salient part of their identity in the workplace, which is strongly contextually dependent, for idealistic activists). (2) The disclosure dilemma, to account for the particular workplace experiences of those possessing an invisible stigma (e.g., sexual minorities). This may either be an individual dilemma, as proposed by extant research literature, or a shared dilemma, a notion for which we found some preliminary indications. (3) The distinction of office versus field context, strongly impacting the salience and importance of each of the contextual characteristics.

Figure 1 presents an update to Shore et al. (2011) conceptual model, integrating findings from this study. It adds three elements, reflecting the particular context of our case study: individual-level characteristics, the disclosure dilemma, and the focal organizational setting (i.e., office vs. field). Additionally, Table 1 presents our findings, delineating the three inductively constructed groups, the defining individual-level characteristics, and each participant’s perceived satisfaction of the needs for authenticity and belonging, based on the interviews.

### Strengths and Limitations

This study drew upon current social and organizational psychological understanding of workplace inclusion to build its theoretical framework, which we complemented with insights from literature on workplace experiences of SGM-employees. Our findings underline the usefulness of this framework by showing how organization-level characteristics can play a role in LGs’ perceptions of workplace inclusion. Importantly, however, it seems that the framework should be further refined, to be able to investigate non-office work settings more carefully, and to enhance its applicability to the lived realities of invisible stigmatized minority groups. We were able to reach a richness within our data due to our chosen method of participant recruitment. In line with our maximum variation sampling strategy, we were able to find commonalities as well as differences among our participants, thereby reaching a satisfactory level of data saturation (see e.g. Hennink et al., 2011). These commonalities and differences formed the basis for our conceptual analyses, providing us with new insights into the central topics of this study. Furthermore, we recruited people through the gatekeeper strategy, thereby guaranteeing a certain degree of self-selection. This, then, translated into interviewing participants who were willing to share insightful stories about their personal experiences.

Simultaneously, this self-selection procedure, might have created a considerable amount of bias, and, thus, be considered a limitation of the study. Perhaps the employees who participated in our study were the ones who are particularly interested in sharing their stories, thereby making it possible that we did not get to speak with the less vocal segment of MSF’s SGM-employees. Similarly, a segment of MSF’s SGM-population may not have been able to live with a lowered satisfaction of their need for authenticity, and therefore decided to switch to another job. Subsequently, we were not able to collect insights into the factors leading to turnover decisions. Another limitation is that our sample only consisted of LG humanitarian aid workers. Given the particular issues faced by bisexual populations (e.g., lowered mental and emotional wellbeing, and social support compared to LGs; Arena and Jones, 2017), and transgendered people (e.g., more severe employment discrimination and unique challenges in identity management; Connell, 2010), it may be very likely that their experiences differ from the ones described here.

## Avenues for Future Research

Based on the findings we present here, we suggest several fruitful avenues for future research: Two of a theoretical, one of a methodological, and two of a practical nature. A first theoretical idea hinges on our finding of the possible existence of a *shared* disclosure dilemma, rather than the commonly assumed individual dilemma (e.g., Griffith and Hebl, 2002). For example, future studies could explore the interests and viewpoints of the multiple stakeholders involved in the disclosure dilemma, the particular contexts in which such a shared dilemma may emerge, and what consequences may arise out of such ‘conflicts of interest,’ for individual and organization alike.

A second theoretical idea relies on the possibility of a buffering effect among the needs that combine to conceptualize workplace inclusion, for example by looking into how applicable the current conceptualization is for those people who have the option to conceal a characteristic (e.g., religion, educational background, political orientation) that distinguishes them from the majority. How does (lack of) satisfaction of their need for authenticity affect their perceived inclusion? Furthermore, in this particular case study, the organization was lauded by its ability to nurture perceptions of belonging, thereby somewhat ‘lessening the blow’ of lowered fulfillment of their need for authenticity. Do SGM-employees and fellow ‘invisible’ minorities in other sectors also experience that their need for authenticity needs to be compromised in their daily work, but that fulfillment of their belonging need may have a buffering function, or vice versa? What role does identity centrality play in the decision to disclose an invisible identity, and how does it affect perceived workplace inclusion?

Addressing these questions may lead to uncovering the conditions under which a sexual minority employee’s different social identities become salient in a particular setting. For example, findings of our case study indicate the importance of an overarching organizational goal, which made employees subsume their personal need for authenticity under the organizational needs, especially in the field.

With regard to methods, our study calls for replication in other contexts. It might be possible that humanitarian aid work, with its high volatility, stress-inducing workload, and extreme ability to foster a strong sense of belonging, may be a very special organizational setting, leading to the findings we presented here. Additionally, there is a strong self-selection in this type of profession; as several participants mentioned, you have to be a certain kind of individual to be willing to live under these very special circumstances. This may have influenced our findings. Therefore, future research would do well to investigate these components of workplace inclusion within other organizations, in particular those with a strong mission element and the aim to help or save others, such as the ICRC, the military, volunteering, or faith-based organizations.

Thirdly, two more practical areas deserve attention. As the office setting of our study is situated in Amsterdam, we were not surprised to hear that, compared to other countries, participants felt they could be open about their sexual identity, hinging on the image of Amsterdam, and the Netherlands at large, to be tolerant and open toward SGM. Nevertheless, we also found boundaries to this openness: participants indicated to still be the subject of, or having witnessed someone else being the subject of, derogatory jokes and remarks toward SGM. We need to disentangle the mechanisms through which this ‘bounded tolerance’ (see also Buijs et al., 2011) strains SGM-employees, how negative consequences can be overcome, and how this can be addressed among heterosexual workers. Relatedly, we only considered the viewpoints of LG employees. A truly inclusive work environment also needs to take into consideration the viewpoints of the majority group (i.e., heterosexuals) (cf. Shore et al., 2011; Otten and Jansen, 2015), and of other SGM, namely bisexuals’, and transgender individuals’.

## Conclusion

We found similarities as well as differences among sexual minority humanitarian aid workers, for example with regard to how they assess the disclosure dilemma, and with regard to their perceived workplace inclusion. This suggests that, even if a fundamental human need cannot be satisfied within a given organizational environment, sexual minority employees might continue to enjoy working for their organization, because of a deep love and great passion for their job, and for fulfilling an overarching organizational mission. There are ways in which they can make meaningful contributions, both within and beyond their organizational boundaries, even though it may be more difficult to fulfill their need for authenticity within the workplace, compared to members of the majority group. One way to better understand the mechanisms that perpetuate existing organizational arrangements maintaining workplace inequality of vulnerable groups, such as sexual minorities, is to do in-depth research on workplace inclusion from the targets’ own perspectives.

## Data Availability

The dataset for this manuscript is not publicly available because of the sensitive information it contains with regard to participants’ personal experiences related to their sexual identity, and because of the confidential information it contains on Médecins Sans Frontières. Requests to access the dataset should be directed to Julian Rengers, j.m.rengers@rug.nl.

## Author Contributions

JR and LH established the conceptual framework and research design of this study. JR conducted the interviews, analyzed the data, and wrote the manuscript. LH, SO, and RW provided feedback on and contributed to rewriting of the manuscript. All authors approved the final version of this manuscript.

## Conflict of Interest Statement

The authors declare that the research was conducted in the absence of any commercial or financial relationships that could be construed as a potential conflict of interest.

## Footnotes

1 Most often, international staff members originate from Westernized countries, such as Western European countries, Canada, United States, or Australia. However, several participants noticed a recent tendency to hire more international staff members from countries that traditionally have been the target of their work.

2 A full list of project countries in which MSF-OCA is at work can be found at https://www.artsenzondergrenzen.nl/projecten.

3 The gender with which the participant identifies, with F indicating female and M indicating male.

4 Referring to people whose gender identity and expression matches their assigned sex at birth

5 Generally, this demeanor was far more negative in the field. For example, one participant recalled that “of course, there’s a lot of gay jokes […] if you’re a bit lame, they always call you ‘ah, you’re a gay, you can’t lift that”’ (M, 7½ years’ experience in office and field).

6 Within the field, there are different supervisor roles. Examples include the field coordinator (in charge of implementation and daily management of the project), head of project (carrying final responsibility over a subproject within the mission), and head of mission (carrying final responsibility over the whole mission).

## References

[B1] Arena D. F. Jr. Jones K. P. (2017). To “B” or not to “B”: assessing the disclosure dilemma of bisexual individuals at work. *J. Vocat. Behav.* 103 86–98. 10.1016/j.jvb.2017.08.009

[B2] Baumeister R. F. Leary M. R. (1995). The need to belong: desire for interpersonal attachments as a fundamental human motivation. *Psychol. Bull.* 117 497–529. 10.1037/0033-2909.117.3.497 7777651

[B3] Brewer M. B. (1991). The social self: on being the same and different at the same time. *Pers. Soc. Psychol. Bull.* 17 475–482. 10.1177/0146167291175001

[B4] Buijs L. Hekma G. Duyvendak J. W. (2011). ‘As long as they keep away from me’: the paradox of antigay violence in a gay-friendly country. *Sexualities* 14 632–652. 10.1177/1363460711422304

[B5] Button S. B. (2001). Organizational efforts to affirm sexual diversity: a cross-level examination. *J. Appl. Psychol.* 86 17–28. 10.1037/0021-9010.86.1.17 11302229

[B6] Button S. B. (2004). Identity management strategies utilized by lesbian and gay employees. A quantitative investigation. *Group Organ. Manag.* 29 470–494. 10.1177/1059601103257417

[B7] Chaudoir S. R. Fisher J. D. (2010). The disclosure processes model: understanding disclosure decision-making and post-disclosure outcomes among people living with a concealable stigmatized identity. *Psychol. Bull.* 136 236–256. 10.1037/a0018193 20192562 PMC2922991

[B8] Chrobot-Mason D. Button S. B. DiClementi J. D. (2001). Sexual identity management strategies: an exploration of antecedents and consequences. *Sex Roles* 45 321–336. 10.1023/A:1014357514405

[B9] Clair J. A. Beatty J. E. MacLean T. L. (2005). Out of sight, but not out of mind: managing invisible social identities in the workplace. *Acad. Manag. Rev.* 30 78–95. 10.5465/AMR.2005.15281431

[B10] Colgan F. Creegan C. McKearney A. Wright T. (2007). Equality and diversity policies and practices at work: lesbian, gay and bisexual workers. *Equal Opport. Int.* 26 590–609. 10.1108/02610150710777060

[B11] Colgan F. McKearney A. (2012). Visibility and voice in organisations: lesbian, gay, bisexual and transgendered employee networks. *Equal. Div. Inclus.* 31 359–378. 10.1108/02610151211223049

[B12] Colgan F. Wright T. (2011). Lesbian, gay and bisexual equality in a modernizing public sector 1997–2010: opportunities and threats. *Gend. Work Organ.* 18 548–570. 10.1111/j.1468-0432.2011.00558.x

[B13] Connell C. (2010). Doing, undoing, or redoing gender? Learning from the workplace experiences of transpeople. *Gend. Soc.* 24 31–55. 10.1177/0891243209356429

[B14] Corbin J. Strauss A. L. (2008). *Basics of Qualitative Research: Techniques and Procedures for Developing Grounded Theory.* Thousand Oaks, CA: SAGE Publications Ltd 10.4135/9781452230153

[B15] Cramwinckel F. M. Scheepers D. Van der Toorn J. (2018). Interventions to reduce blatant and subtle sexual orientation- and gender identity prejudice (SOGIP): current knowledge and future directions. *Soc. Issues Policy Rev.* 12 183–217. 10.1111/sipr.12044

[B16] Crocker J. Major B. Steele C. (1998). “Social stigma,” in *The Handbook of Social Psychology* eds Gilbert D. T. Fiske S. T. Lindzey G. (New York, NY: McGraw Hill) 504–553.

[B17] Croteau J. M. (1996). Research on the work experiences of lesbian, gay, and bisexual people: an integrative review of methodology and findings. *J. Vocat. Behav.* 48 195–209. 10.1006/jvbe.1996.0018

[B18] Deci E. L. Ryan R. M. (2000). The “what” and “why” of goal pursuits: human needs the self-determination of behavior. *Psychol. Inquiry* 11 227–268. 10.1207/S15327965PLI1104_01 27055568

[B19] Ellemers N. Barreto M. (2006). Social identity and self-representation at work: how attempts to hide a stigmatized identity affect emotional well-being, social inclusion and performance. *Neth. J. Psychol.* 62 51–57. 10.1007/BF03061051

[B20] Ellemers N. Jetten J. (2013). The many ways to be marginal in a group. *Pers. Soc. Psychol. Rev.* 17 3–21. 10.1177/1088868312453086 22854860

[B21] Eriksson C. B. Bjorck J. P. Larson L. C. Walling S. M. Trice G. A. Fawcett J. (2009). Social support, organisational support, and religious support in relation to burnout in expatriate humanitarian aid workers. *Ment. Health Relig. Cult.* 12 671–686. 10.1080/13674670903029146

[B22] Friese S. (2013). *ATLAS.ti 7 User Manual.* Berlin: ATLAS.ti Scientific Software Development GmbH.

[B23] Goffman E. (1959). *The Presentation of Self in Everyday Life.* New York, NY: Anchor Books.

[B24] Goffman E. (1963). *Stigma. Notes on the Management of Spoiled Identity.* Upper Saddle River, NJ: Prentice Hall.

[B25] Griffith K. H. Hebl M. R. (2002). The disclosure dilemma for gay men and lesbians: “Coming out” at work. *J. Appl. Psychol.* 87 1191–1199. 10.1037/0021-9010.87.6.1191 12558225

[B26] Guba E. G. (1981). Criteria for assessing the trustworthiness of naturalistic inquiries. *Educ. Commun. Technol. J.* 29 75–91.

[B27] Hennink M. Hutter I. Bailey A. (2011). *Qualitative Research Methods.* London, UK: Sage Publications.

[B28] ILGA (2016). *The ILGA-RIWI 2016 Global Attitudes Survey on LGBTI People in Partnership with LOGO.* Available at: http://ilga.org/global-survey-attitudes-lgbti-riwi-logo/

[B29] ILGA (2017). *Sexual Orientation Laws in the World – Overview.* Available at: http://ilga.org/downloads/2017/ILGA_WorldMap_ENGLISH_Overview_2017.pdf

[B30] Jansen W. S. Otten S. Van der Zee K. Jans L. (2014). Inclusion: conceptualization and measurement. *Eur. J. Soc. Psychol.* 44 370–385. 10.1002/ejsp.2011

[B31] Jansen W. S. Vos M. W. Otten S. Podsiadlowski A. Van der Zee K. I. (2016). Colorblind or colorful? how diversity approaches affect cultural majority and minority employees. *J. Appl. Soc. Psychol.* 46 81–93. 10.1111/jasp.12332

[B32] Janssens M. Zanoni P. (2008). “What makes an organization inclusive? Organizational practices favoring the relational inclusion of ethnic minorities in operative jobs. *Paper presented at the IACM 21st Annual Conference on Social Science Research Network* Chicago, IL. 10.2139/ssrn.1298591

[B33] Jones K. P. King E. B. (2014). Managing concealable stigmas at work: a review and multilevel model. *J. Manag.* 40 1466–1494. 10.1177/0149206313515518

[B34] Kernis M. H. Goldman B. M. (2006). “A multicomponent conceptualization of authenticity: theory and research,” in *Advances in Experimental Social Psychology* ed. Zanna M. P. (San Diego, CA: Elsevier Academic Press) 283–357.

[B35] King E. B. Mohr J. J. Peddie C. I. Jones K. P. Kendra M. (2017). Predictors of identity management: an exploratory experience-sampling study of lesbian, gay, and bisexual workers. *J. Manag.* 43 476–502. 10.1177/0149206314539350

[B36] Lloren A. Parini L. (2016). How LGBT-supportive workplace policies shape the experience of lesbian, gay men, and bisexual employees. *Sex. Res. Soc. Policy* 14 289–299. 10.1007/s13178-016-0253-x

[B37] McFadden C. (2015). Lesbian, gay, bisexual, and transgender careers and human resource development: a systematic literature review. *Hum. Res. Dev. Rev.* 14 125–162. 10.1177/1534484314549456

[B38] Médecins Sans Frontières (2017). *Annual Report MSF-OCA 2017.* Available at: https://www.artsenzondergrenzen.nl/jaarverslag

[B39] Médecins Sans Frontières (2018). *The MSF Charter.* Available at: https://www.msf.org/who-we-are

[B40] Mor Barak M. E. Cherin D. (1998). A tool to expand organizational understanding of workforce diversity. *Adm. Soc. Work* 22 47–64. 10.1300/J147v22n01_04

[B41] Nembhard I. M. Edmondson A. C. (2006). Making it safe: the effects of leader inclusiveness and professional status on psychological safety and improvement efforts in health care teams. *J. Organ. Behav.* 27 941–966. 10.1002/job.413

[B42] Ng E. S. Rumens N. (2017). Diversity and inclusion for LGBT workers: current issues and new horizons for research. *Can. J. Adm. Sci.* 34 109–120. 10.1002/CJAS.1443

[B43] Nishii L. H. (2013). The benefits of climate for inclusion for gender-diverse groups. *Acad. Manag. J.* 56 1754–1774. 10.5465/amj.2009.0823

[B44] Orne J. (2011). ‘You will always have to “out” yourself’: reconsidering coming out through strategic outness. *Sexualities* 14 681–703. 10.1177/1363460711420462

[B45] Otten S. Jansen W. S. (2015). “Predictors and consequences of exclusion and inclusion at the culturally diverse workplace,” in *Towards Inclusive Organizations. Determinants of successful diversity management at work* eds Otten S. Van der Zee K. I. Brewer M. B. (Hove: Psychology Press) 67–86.

[B46] Ozeren E. (2014). Sexual orientation discrimination in the workplace: a systematic review of literature. *Proc. Soc. Behav. Sci.* 109 1203–1215. 10.1016/j.sbspro.2013.12.613

[B47] Pachankis J. (2007). The psychological implications of concealing a stigma: a cognitive-affective-behavioral model. *Psychol. Bull.* 133 328–345. 10.1037/0033-2909.133.2.328 17338603

[B48] Patton M. Q. (2001). *Qualitative Research and Evaluation Methods.* Thousand Oaks, CA: Sage Publications.

[B49] PEW Research Center (2013). *The Global Divide on Homosexuality: Greater Acceptance in More Secular and Affluent Countries.* Available at: http://www.pewglobal.org/2013/06/04/the-global-divide-on-homosexuality/

[B50] Priola V. Lasio D. De Simeone S. Serri F. (2014). The sound of silence: lesbian, gay, bisexual and transgender discrimination in ‘inclusive organizations.’ *Br. J. Manag.* 25 488–502. 10.1111/1467-8551.12043

[B51] Ragins B. R. (2004). Sexual orientation in the workplace: the unique work and career experiences of gay, lesbian and bisexual workers. *Res. Pers. Hum. Res. Manag.* 23 37–122. 10.1016/S0742-7301(04)23002-X

[B52] Ragins B. R. (2008). Disclosure disconnects: antecedents and consequences of disclosing invisible stigmas across life domains. *Acad. Manag. Rev.* 33 194–215. 10.2307/20159383

[B53] Ragins B. R. Cornwell J. M. (2001). Pink triangles: antecedents and consequences of disclosing invisible stigmas across life domains. *J. Appl. Psychol.* 86 1244–1261. 10.1037//0021-9010.86.6.1244 11768065

[B54] Ragins B. R. Singh R. Cornwell J. M. (2007). Making the invisible visible: fear and disclosure of sexual orientation at work. *J. Appl. Psychol.* 92 1103–1118. 10.1037/0021-9010.92.4.1103 17638468

[B55] Roberson Q. M. (2006). Disentangling the meanings of diversity and inclusion in organizations. *Group Organ. Manag.* 31 212–236. 10.1177/1059601104273064

[B56] Saldaña J. (2009). *The Coding Manual for Qualitative Researchers.* London: Sage Publications.

[B57] Settles I. H. (2004). When multiple identities interfere: the role of identity centrality. *Pers. Soc. Psychol. Bull.* 30 487–500. 10.1177/0146167203261885 15070477

[B58] Shenton A. K. (2004). Strategies for ensuring trustworthiness in qualitative research projects. *Edu. Inform.* 22 63–75. 10.3233/EFI-2004-22201

[B59] Shore L. M. Randel A. E. Chung B. G. Dean M. A. Holcombe Ehrhart K. Singh G. (2011). Inclusion and diversity in work groups: a review and model for future research. *J. Manag.* 37 1262–1289. 10.1177/0149206310385943

[B60] Spiegel T. Wittek R. Steverink N. (2016). What are the pathways linking the disclosure of a degenerative eye condition in the workplace and wellbeing? a mixed methods approach. *Int. J. Dis. Manag.* 11 1–12. 10.1017/idm.2016.2

[B61] Sue D. W. (2010). *Microaggressions in Everyday Life: Race, Gender, and Sexual Orientation.* Hoboken, NJ: Wiley.

[B62] Turner J. C. Hogg M. A. Oakes P. J. Reicher S. D. Wetherell M. S. (1987). *Rediscovering the Social Group: A Self-Categorization Theory.* Oxford: Blackwell.

[B63] Visser M. Mills M. Heyse L. Wittek R. P. M. (2016). Work-life balance among humanitarian aid workers. *Nonprofit Volunt. Sect. Q.* 45 1191–1213. 10.1177/0899764016634890

[B64] Wax A. Coletti K. K. Ogaz J. W. (2018). The benefit of full disclosure: a meta-analysis of the implications of coming out at work. *Organ. Psychol. Rev.* 8 3–30. 10.1177/2041386617734582

[B65] Webster J. R. Adams G. A. Maranto C. L. Sawyer K. Thoroughgood C. (2017). Workplace contextual supports for LGBT employees: a review, meta-analysis, and agenda for future research. *Hum. Res. Manag.* 57 1–18. 10.1002/hrm.21873

