# Supplementary material for: “It’s Not Always Possible to Live Your Life Openly or Honestly in the Same Way” – Workplace Inclusion of Lesbian and Gay Humanitarian Aid Workers in Doctors Without Borders
Source: Front Psychol. 2019 Feb 27;10:320. doi: 10.3389/fpsyg.2019.00320 (PMC6400840; doi:10.3389/fpsyg.2019.00320)
Supplement: Supplementary file 1 [file Table_1.DOCX]

“It’s not always possible to live your life openly or honestly in the same way” – Workplace inclusion of lesbian and gay humanitarian aid workers in Doctors without Borders

Julian Rengers, Liesbet Heyse, Sabine, Otten, and RafaelWittek

Categories and subcategories in the codebook

1. **Inclusiveness climate**
   1. *attitudes colleagues*
   2. *contact with colleagues*
   3. *cooperation with colleagues*
   4. *support from colleagues*
2. **Inclusive leadership**
   1. *attitudes supervisors*
   2. *contact with colleagues*
   3. *support from supervisors*
3. **Inclusiveness practices**
   1. *diversity and equality*
      1. *(queer) invisibility*
   2. *policy*
   3. *awareness*
      1. *debate*
   4. *barrier*
   5. *facilitation*
      1. *initiatives*
      2. *start*
      3. *agenda*
   6. *façade*
   7. *organizational culture*
   8. *organizational support*
      1. *lack of organizational support*
4. **Disclosure dilemma**
   1. *heteronormativity*
   2. *fear of disclosure*
   3. *degree of openness*
   4. *outed*
   5. *stress*
5. **Belonging**
   1. *thick description*
   2. *lack of belonging*
6. **Authenticity**
   1. *thick description*
   2. *lack of authenticity*
7. **Context**
   1. *office*
      1. *Rainbow Network*
         1. *questionnaire*
         2. *activities*
         3. *role*
   2. *field*
      1. *cultural framework*
      2. *legal framework*
      3. *expat status*
      4. *(queer) community*
         1. *LGBeneficiaries*
         2. *LGB national staff*
   3. *outside of work*
      1. *support – external*
8. **Discrimination**
   1. *related to MSF*
      1. *homophobia*
   2. *not related to MSF*
9. **Features of the interview**
   1. *clarification*
   2. *doubt*
10. **Information on the organization**
    1. *briefing*
    2. *image*
    3. *interorganizational comparison*
    4. *intraorganizational comparison*
    5. *mission*
       1. *principles*
       2. *témoignage*
    6. *patronizing*
    7. *research*
    8. *association*
11. **Personal information**
    1. *background information*
       1. *age*
       2. *country of origin*
       3. *education*
       4. *family life*
       5. *gender*
       6. *living situation*
       7. *married*
          1. *not married*
       8. *residence*
       9. *tenure*
    2. *personal beliefs, perspectives, opinions, and ideas*
       1. *consideration*
       2. *experience*
       3. *future*
       4. *motivation*
       5. *respect*
       6. *responsibility*
       7. *shared experience*
       8. *unclearness*
       9. *valence*
          1. *positive*
          2. *negative*
    3. *about the job*
       1. *personal safety*
       2. *strategy*
       3. *team*
       4. *understanding*
       5. *work*
          1. *current*
          2. *history*
          3. *characteristic*
