# Supplementary material for: “It’s Not Always Possible to Live Your Life Openly or Honestly in the Same Way” – Workplace Inclusion of Lesbian and Gay Humanitarian Aid Workers in Doctors Without Borders
Source: Front Psychol. 2019 Feb 27;10:320. doi: 10.3389/fpsyg.2019.00320 (PMC6400840; doi:10.3389/fpsyg.2019.00320)
Supplement: Supplementary file 2 [file Table_2.DOCX]

A thick description of the code ‘Authenticity’ in the office context

| **What are the different aspects?** | **What is the context and meaning?** | **How is each aspect discussed?** | **How often?** | **What other codes intersect?** | **Dimension** |
| --- | --- | --- | --- | --- | --- |
| Sharing personal stories | Being as open in sharing information about one’s personal life as straight co-workers; when they talk, I talk, and when they ask, I answer | Comfortable; open; supportive; should be possible | Mentioned most often | Attitude(s) – colleague(s); Attitude(s) – supervisor(s) | Individual |
| Similarities to straights | We are similar to straight individuals (e.g., we want equal treatment with regard to family policies) | Individual but similar; emphasis on similarities; we are like them but with this particular situation | Mentioned by few | Equality | Individual |
| Feel welcomed | The organization makes everyone feel welcome, and gives the message that it’s okay to be who you are | Every personality is appreciated; focus on strengths, not weaknesses; MSF tries hard to create a sense of inclusivity | Frequently mentioned | Organizational culture | Organizational |
| Human right | Participants feel that they have the right to be open about their sexual identity | It’s the right thing to do; it’s my right to be who I am | Mentioned by some | - | Individual |
| Multiple identities | Participants are not defined by just their sexual identity; it is but one part of who you are as a person | Inextricable part of me, but nothing more than that | Mentioned by some | - | Individual |
| Building interpersonal relationship | It is important to be as open about who you are as you can be, if you want to build social relationships with colleagues | Making others comfortable with SGM; create trust; do not want to create a lie | Frequently mentioned | Contact – colleagues; Contact – supervisors | individual |
| Trainings | Managers should be taught tools as to how to deal with a diverse workforce | Should be on managerial agenda; make issues open for discussion; to get people thinking about it | Frequently mentioned | Organizational support; Initiatives | Organizational |
| That’s just the way it is | There’s nothing more to it; we’re also human, | People are who they are; I just am what I am | Mentioned by some | - | Individual |
| Heterosexuals’ responsibilities | Straight co-workers also have the responsibility to create room and space to discuss sexuality issues | We have to fight for every inch; hoping to happen more organically | Mentioned by few | Attitude(s) – colleague(s); Attitude(s) – supervisor(s) | Organizational |
| Organizational responsibilities | The organization should visibly show support for this community | Visible support; when you have people in power [showing support], it starts to trickle down | Frequently mentioned | Responsibility;  Organizational support | Organizational |
| Raising awareness by being open | By being open about one’s sexual identity, participants feel they raise awareness about the 1) presence of SGM in MSF, and 2) their particular issues | With awareness you create understanding; | Mentioned by some | Visibility; Awareness | Individual |

A thick description of the code ‘Authenticity’ in the field context

| **What are the different aspects?** | **What is the context and meaning?** | **How is each aspect discussed?** | **How often?** | **What other codes intersect?** | **Dimension** |
| --- | --- | --- | --- | --- | --- |
| Living a closeted life | In the field, participants cannot be open about their sexuality and therefore go back ‘into the closet’ | Uncomfortable; deny aspects of personal life | Very often | Lack of authenticity; Disclosure dilemma | Individual |
| *Being forced* to live a closeted life | The organization does not offer any other option than to live a closeted life in the field; the modus operandi is Don’t Ask Don’t Tell | Forced; lack of choice; should be personal decision; this should not be like this; don’t want to be cautious | Most often | Lack of authenticity; Lack of organizational support | Organizational |
| Length of mission | The longer your mission lasts, the more difficult it is to not be your authentic self | More difficult to see partner and family (e.g., Skype); no possibility for partner to come over | Frequently mentioned by those with partner/family | - | Organizational |
| Comparison with others in the field | Comparison with other minority groups (e.g., women, SGM) who live in the project country | Can suck it up for the time being; could be worse; | Mentioned by some | LGBeneficiaries | Individual |
| Work is work, life is life | Strictly separated aspects of one’s life; in the field I work, at home I live | I am really busy, because the work is very demanding; you are not there to make personal relationships | Mentioned by some | Mission; | Individual |
| Particular identity management strategies | To indicate one’s sexual identity to others, one can engage in multiple strategies (e.g., signaling, fabricating lies, normalizing) | Playing around with it; no general announcement; open if context is right; describing rather than saying | Mentioned by few | Disclosure dilemma; | Individual |
| Role model | Being a role model for others in being out on a mission, to show others being LG and a humanitarian aid worker can be combined | If I am out, I can help others be out; have to be activist; as long as it continues to be a deep dark secret, nobody’s mind gets changed | Mentioned by few | - | Individual |
| Respect circumstances | We have to have respect for the circumstances in which we work | Accept and adjust | Mentioned by some | Cultural framework;  Legal framework | Organizational |

A thick description of the code ‘Belonging’ in the context of this study

| **What are the different aspects?** | **What is the context and meaning?** | **How is each aspect discussed?** | **How often?** | **What other codes intersect?** | **Dimension** |
| --- | --- | --- | --- | --- | --- |
| Always a member | Participants always feel like they are a member of the organization (i.e., group membership) | On weekends, during free time; I *am* part of this organization; every day, every moment; never not part of it; when signing up, it’s not just a job; it’s a big part of my life | Most frequently mentioned | - | Individual |
| Even in critical situations | Even though there are critical moments when you feel less part of the organization, you will always feel a member of this organization | There are moments when you feel a bit alone […] the organization made me feel like you’re part of it; even if I had bad experiences, I never felt that I could never represent this organization anymore | Mentioned by some | Lack of organizational support | individual |
| Even stronger membership in the field | Participants feel even more strongly a member of MSF when they are in the field, directly contributing to fulfilling its organizational mission | When you’re in the field, wearing the shirt; seeing what we do; directly contribute to aims; when we’re providing really strong services | Frequently mentioned | Field | Individual |
| Organizational efforts | The organization tries hard to create a shared feeling from the moment you join (e.g., training days, organizational artefacts, image) | The moment you join, you feel at home; tried hard to create that sense of inclusivity; MSF has very much a sort of family feel to it | Mentioned by few | Initiatives | Organizational |
| All together | When participants realize they are all working towards a larger, common goal, they feel strong belonging with the organization | We’re here for the same reasons; there’s very much a sort of family feel to it; you’re part of the machine | Frequently mentioned | - | Organizational |
| Attention from outside | When participants notice that others are interested in their work at MSF, because of the organizational image MSF has | It’s *my* organization; media coverage; during birthday parties, people ask, because MSF is interesting | Mentioned by few | - | Organizational |
| Pride | Participants experience a feeling of pride when it comes to their organization | I feel proud to be part of this organization; proud of representing | Mentioned by some | - | Individual |
| Identification with organizational principles | Participants feel a member of the organization because they can see their own principles and values reflected in the organization’s principles | I have the same vision; I can see myself in that; shared principles; I identify myself with MSF | Mentioned by some | Principles | Individual |

NB. We decided against creating a separate table detailing the differences of perceived belonging in either the office or the field context, because there are many similarities between these two. Because participants feel almost unanimously and unequivocally that they are a member of the organization, there is little variation. The only additional aspect in the field is that this belonging is even stronger there, because participants are directly contributing to the organizational aims. Nevertheless, we did not deem this sufficient to be presented in a separate table.
