# Supplementary material for: “It’s Not Always Possible to Live Your Life Openly or Honestly in the Same Way” – Workplace Inclusion of Lesbian and Gay Humanitarian Aid Workers in Doctors Without Borders
Source: Front Psychol. 2019 Feb 27;10:320. doi: 10.3389/fpsyg.2019.00320 (PMC6400840; doi:10.3389/fpsyg.2019.00320)
Supplement: Supplementary file 3 [file Table_3.DOCX]

| **Element** | **Question(s)** | **Explanation** |
| --- | --- | --- |
| 1. **Inclusiveness climate** | *How do you experience working for MSF?* (4);  *How would you describe the contact you have with your colleagues?* (7) | Addresses the participant’s assessment of the positive and/or negative aspects of working for MSF, and could provide some insights into the perceived openness or hostility of the organizational climate;  Encompasses the participant’s colleagues’ openness towards him/her as an LGBQ individual. |
| 2. **Inclusive leadership** | *And how would you describe the contact you have with your supervisor(s)?* (8) | Encompasses the relationship between participant and supervisor(s). Might indicate openness towards him/her as an LGBQ individual. |
| 3. **Inclusiveness practices** | *What do you think MSF-OCA could do to support LGBQ staff members within the office?* (17);  *What do you think MSF-OCA could do to support LGBQ staff members during field missions?* (18) | Both questions address the participant’s thoughts and ideas on how the organization could facilitate the work of LGBQ staff members; by analysing what is said, I can deduce which practices are currently *not* in place. |
| 4. **Disclosure dilemma** | *Why did you decide to (not) be open about your sexual orientation?* (13a/b, after previously asking how open they are about their sexual orientation) | Demonstrates one’s reasons and potential struggles in dealing with their sexual identity in the workplace. |
| 5a. **Belonging** | *Could you tell me about moments when you feel very much a member of MSF?* (9);  *And could you tell me about moments when you do not feel very much a member of MSF?* (10);  *Could you tell me about some of your experiences as an LGBQ individual working for MSF?* (14) | Together, these address one’s degree to which s/he feels like s/he belongs to MSF, and is able to have stable and positive relations within the organization;  Open question which could show examples of feeling high or low in belonging. |
| 5b. **Authenticity** | *Could you tell me about some of your experiences as an LGBQ individual working for MSF?* (14) | Open question which could show examples of feeling high or low in authenticity. |

“It’s not always possible to live your life openly or honestly in the same way” – Workplace inclusion of lesbian and gay humanitarian aid workers in Doctors without Borders

Julian Rengers, Liesbet Heyse, Sabine Otten, and Rafael Wittek

Operationalizations of the core concepts of the conceptual model
