# Supplementary material for: “It’s Not Always Possible to Live Your Life Openly or Honestly in the Same Way” – Workplace Inclusion of Lesbian and Gay Humanitarian Aid Workers in Doctors Without Borders
Source: Front Psychol. 2019 Feb 27;10:320. doi: 10.3389/fpsyg.2019.00320 (PMC6400840; doi:10.3389/fpsyg.2019.00320)
Supplement: Supplementary file 4 [file Table_4.DOCX]

“It’s not always possible to live your life openly or honestly in the same way” – Workplace inclusion of lesbian and gay humanitarian aid workers in Doctors without Borders

Julian Rengers, Liesbet Heyse, Sabine Otten, and Rafael Wittek

This codebook presents an overview of all codes that were applied in this study. Codes have been developed deductively and inductively, in accordance with guidelines proposed by grounded theory (Hennink et al., 2011). Three potential ways in which codes can be developed deductively are based on either the interview guide, current research literature, or personal or professional experience of the researcher (see Hennink et al., 2011, p. 219). Moreover, there are several ways in which codes can be developed inductively. These include reading for overall content, annotating data, noticing repetition, and by analytic reading. The codebook contains relevant information of each individual code, including the name of the code, the type of code (i.e., deductive or inductive) as well as the strategy through which this code came to being, a description of the code to indicate to which segments of speech it should be applied, as well as an indication to which segments it should not be applied if necessary, and, finally, an example as collected from the data.

| **Code** | **Type** | **Description** | **Example(s) from data** |
| --- | --- | --- | --- |
| *Activities* | Inductive, read for overall content (OC) | Apply when the participant discusses activities undertaken by the Rainbow Network. | “Even in the Rainbow Network we have some social activities.”  “Participate in Pride Walk when it’s Pride Week here in Amsterdam.” |
| *Age* | Deductive, interview guide (IG) | The participant’s age. | “38. Yeah, I’m 38.” |
| *Agenda* | Inductive, notice repetition (NR) | Apply to instances of speech in which the participant mentions that they think that something should be on MSF’s agenda. | “This is something that I would like to see addressed readily by the senior management.”  “Bring the topic up.” |
| *Anecdote* | Inductive, analytic reading (AR) | Apply to situations when the participant attempts to illustrate what they intend to say through the use of an anecdote. Also apply when the participant shares a personal story, not necessarily directly related to the research topic at hand. | “For example, maybe two years ago, we were hiring a colleague, and I said: ‘Okay, look guys; there’s four twenty something white females in your team, perhaps you might want to think about male or non-white, non-Dutch speaker within the team who may speak French or Arabic, which is becoming more prevalent in our work.’” |
| *Association* | Inductive, OC | Apply when the participant refers to the nature of MSF, being an association or a movement. | “So I stayed involved with MSF through the association.” |
| *Assumption* | Inductive, OC | This code is used when the participant mentions assumed heterosexuality of every individual. | “Well, don’t immediately assume that everyone is heterosexual.” |
| *Attitude(s) – colleague(s)* | Deductive, research literature (RL) | Use when the participant mentions colleagues’ attitudes towards either themselves as an LGBQ individual or towards LGBQ individuals in general. Can be used to more closely examine the organization’s inclusiveness climate. | “…and at least the ones who know who I am, yeah, they’re very open to it.”  “…people have come up to me afterwards and said ‘That was so refreshing. That was amazing that you were just so open.’” |
| *Attitude(s) – supervisor(s)* | Deductive, RL | Use when the participant mentions supervisors’ attitudes towards either themselves as an LGBQ individual or towards LGBQ individuals in general. Can be used to more closely examine the organization’s inclusiveness climate, as well as its inclusive leadership. | “My first supervisor, he asked me ‘So, are you married?’ and I was trying to start, because he opened the conversation about it, and then on a certain point, he interrupted me and he said ‘Oh, I don’t want to hear it.’”  “He’s one of the best persons that I work with, and he’s also a superior, and in spite of it all, he has been very open to me and my partner” |
| *Authenticity* | Deductive, RL | One of the two components of the psychological experience of inclusion, defined as the need to stay true to oneself. Apply when the participant mentions moments in which s/he perceived the organization to enable acting in accordance with who s/he truly is. | “A sort basic idea that I think I should be open, and that I strive for that as much as possible, and that I’d like to have as few issues around that topic as possible, because it’s only a small part of your identity.”  “If they talk about their partner, I also talk about my partner. Like that.” |
| *Awareness* | Inductive, NR | Apply when the participant mentions the awareness, consciousness or sensitivity among co-workers and the organization of the importance and salience of the topic of sexual orientation within MSF. | “…they’ve already started certain awareness. And I think that is very important, because, again, for me that’s where it all starts.”  “I see that we can raise awareness. First of all, raise awareness.” |
| *Barrier* | Deductive, professional experience (PrE) | Use when the participant mentions something about a barrier within MSF to being an LGBQ staff member. | “I mean because there’s, like, not a central place? You know, I mean, MSF is just spread out all over the world, I think it makes it a little bit more difficult.” |
| *Belief* | Inductive, AR | Apply when the participant mentions a certain personal belief they hold, which does not necessarily have to be shared by others. | “I think it’s good that it is a topic which is put on the agenda.”  “I just don’t see that it’s relevant at all.” |
| *Belongingness* | Deductive, RL | One of the two components of the psychological experience of inclusion, defined as the need to have stable and recurring interactions with others. Apply when the participant mentions moments in which s/he felt part of the organization. | “I mean any time I see that we’re providing really strong services, and truly fulfilling our mandate.”  “With your T-shirt and the training; you’re immediately immersed in the MSF DNA.”  “I will always feel a part of that, because I *am* a part of that.” |
| *Briefing* | Inductive, OC | When the participant speaks about the briefing or the debriefing, which is the moment when one is being informed about certain things before going on a field mission, or upon returning from the field mission to talk about their experiences. | “For example, you receive a briefing package. So, if you’re going to [country], you receive an entire package with information on [country].”  “I don’t hold back. I never hide it from the expats that I brief or debrief.” |
| *Characteristic* | Inductive, OC | Apply when the participant mentions a specific characteristic of their job at MSF. | “Missions have long-term goals and priorities and immediate goals and priorities.” |
| *Clarification* | Inductive, OC | When the participant asks for clarification of a certain question asked by the interviewer. | “In general, you mean?”  “Sorry, what … did I answer that question?” |
| *Community* | Deductive, PrE | Use when the participant discusses MSF-OCA’s LGBTQ+-community. Also apply when the Rainbow Network (the official name for the LGBTQ+-community within MSF-OCA) is mentioned. | “A sort of icebreaker role, so that they open up the debate, but also a sort of watchdog role, by ensuring that things are going to be implemented, for example in terms of the entire introduction. And that they monitor this as well.” |
| *Considerate* | Inductive, AR | In terms of talking about one’s sexual orientation, when the participant decides to maybe not share as much personal information as they would've liked, in order to prevent other people from being hurt, surprised, or confronted with it. | “Because I just didn’t want to kind of create a problem there.”  “You always have to be careful, because you don’t know how everyone will react to it.” |
| *Contact – colleague(s)* | Deductive, IG | Use when the participant discusses instances in which s/he has contact with (one of) their colleague(s). | “The contact with my direct colleagues is quite open. And direct.” |
| *Contact – supervisor(s)* | Deductive, IG | Use when the participant discusses instances in which s/he has contact with their supervisor(s). | “… who wanted to have a cup of coffee with me to hear how everything was.” |
| *Cooperation* | Inductive, AD | When the participant mentions the cooperation between employees. | “They get that we’re in it together, and we have to find common ground” |
| *Country of origin* | Deductive, IG | The country where the participant is originally from. | “I’m from [country of origin].” |
| *Cultural framework* | Inductive, AR | Since MSF operates in many different cultural contexts and countries, with each of these having their own specific cultural norms and values, intercultural communication or understanding might be difficult, for example between colleagues, or between employee and employer. Apply this code to instances when these culturally derived elements are mentioned. | “When you enter the field, you start to notice that there is a different kind of work relationship. That is very dependent on where you are, because that depends on certain cultural aspects.”  “Depends on the countries that we work in of course, it’s going to have a different impact.”  “That is very difficult because, supposedly, African people do not know how to deal with that!” |
| *Debate* | Inductive, OC | This code is attached to parts of speech when the participant mentions a certain topic that is currently internally debated within MSF. | “Abortion is a big discussion in MSF.”  “To start a discussion about which type of cultural behavior is acceptable in MSF, and which type is not.” |
| *Disclosure dilemma* | Deductive, RL | Apply to segments of speech in which the participant mentions the pros and/or cons of disclosing their non-heterosexuality within the organization. This is not applied when the participant discusses the degree of openness (use *Openness*); rather, it is applied to mark specific reasons for being open or closed about one’s sexual orientation. | “When I went through the next, I would say next and next and next mission, I started to disclose to less and less people, and to be a lot more … secretive about it. Because I start to feel like ‘This is not safe. I don’t feel the organization is backing me up. Nobody’s talking about gay people. Because apparently, we don’t talk about it.’” |
| *Discrimination* | Deductive, RL | Apply to any instance in which the participant mentions discrimination based on their sexual orientation. | “I’ve also been, you know, called names in the street, or by just doing nothing.”  “You can hear the most horrendous jokes in the field, and nobody said ‘This is not accepted in MSF.’” |
| *Diversity* | Inductive, NR | When the participant mentions diversity within MSF, as any of the wide range of dimensions it encompasses. | “The focus is, understandably, very much on cultural diversity, given the type of organization we are. And that is very good. *But* there is also sexual orientation and gender identity and expression, so we want to be part of that as well.” |
| *Doubt* | Inductive, NR | Applied when the participant is uncertain about something. | “Oooh. I don’t know. Eeeeh (laughs). I don’t know. Eeeeeehm.” |
| *Education* | Deductive, IG | Apply when the participant mentions anything related to their education, such as education level, location, field, etc. | “I did like a [education].”  “I’m a holder of [education].” |
| *Equality* | Inductive, AR | When the participant describes the idea of every employee being treated equally, regardless of gender, ethnicity, religion, sexual orientation, or any other characteristic. | “That doesn’t mean that I’m expecting to have a privilege, or special treatment, I want to have the same that everybody, within this individuality that each of us we have, and, you know, to have the same that MSF is offering.” |
| *Expat* | Inductive, NR | When the participant mentions their own status as an expat, either when on a field mission, or in an office setting. | “I can get out of that situation in a short period of time.”  “As an expat, there might be one of you or two of you, and maybe there’s none at a certain point.” |
| *Experience* | Deductive, IG | Apply when the participant discusses how they experience working for MSF. | “It’s very draining work, but there’s nothing else that I really want to do.” |
| *Façade* | Inductive, AR | When the organization or the participant does something to ‘cover up’ a real reason. | “Because it’s not always possible to live your life openly or honestly in the same way.” |
| *Facilitation* | Deductive, PrE | Use when the participant mentions something about facilitating being LGBQ within MSF. | “It so happened that my manager is also gay, so that makes things easier.” |
| *Family life* | Inductive, OC | This code is applied when the participant mentions anything about their family life, for example with their partner, children, or other family members. | “I have two little kids. I don’t see many people outside of working hours.”  “I mean, I have traveled through Uganda with my partner. And, hi! I’m still alive! And nothing has happened to me!” |
| *Fear of disclosure* | Deductive, RL | Apply to any instance in which the participant mentions fear related to their sexual orientation. It is also applied to instances in which fear of disclosing one’s sexual orientation is mentioned, for example due to fearing negative consequences of disclosure. | “Fear of prejudice from my colleagues.”  “So that was also a context I felt a little insecure, so I can only share that with only a certain amount of people I found, yeah, trustworthy.”  “But: anonymous. I didn’t feel comfortable enough to put my name on that.” |
| *Field* | Deductive, PrE | Apply to any instance in which the participant mentions an aspect connected to a field setting. | “When I was in the field, I think basically I *was* back in the closet.” |
| *Future* | Deductive, IG | When the participant discusses their plans for the future. | “Still work for MSF.”  “I hope I can keep working with MSF for a long time.” |
| *Gender* | Deductive, IG | The gender with which the participant identifies. | “Male”  “Female” |
| *Gender differences* | Inductive, OC | When the participant mentions something that might be different for people of different genders. | “It’s for example quite normal to live with two women in a house, you know, so I think you’re generally less visible. So that maybe makes it a bit easier.” |
| *Homophobia* | Inductive, OC | When prejudice against or dislike of homosexual people within MSF is mentioned. | “I’ve always been a little nervous about [country], because it’s just *so* homophobic.” |
| *Image* | Inductive, OC | The organizational image that exists of MSF for outsiders. | “And it’s not the glamorous part, so it’s not what new international people think that all they’re going to be doing is glamorously dodging bullets while saving the life of small smiling black babies. But it’s really more complicated than that.” |
| *Initiatives* | Inductive, OC | This code represents initiatives mentioned by participants that MSF could undertake to contribute to either making the organization more LGBTQ+-friendly, or by contributing to a more positive work environment in the office, or by facilitation/support of the work of sexual minority individuals in the field. | “I think that we should have clear policies or guidelines how we deal with that.”  “An open communication about the fact that there is LGBQ international staff members. So simple acknowledgement.”  “In terms of providing a sort of platform for support, and for trying to kind of move things forward.” |
| *Interorganizational comparison* | Inductive, NR | Is used when MSF, as an organization, is compared to another organization. | “I think that there are places where you can work 8 to 5, and that’s it. And then with MSF, at least for me, you’re still being MSF.” |
| *Intraorganizational comparison* | Inductive, AR | Apply when OCA as a subsection of MSF is compared to other OCs. | “So I found it in OCA more welcoming than in [other OC].” |
| *Lack of authenticity* | Deductive, RL | Apply when the participant mentions moments in which s/he **did not** perceive the organization to enable acting in accordance with who s/he truly is. | “You have to perpetually live a lie.”  “We give up a lot of our personal life.”  “When I feel that the organization is telling me in the back of my mind, maybe it’s silent, or whatever, it’s ‘You don’t belong. You are too special,’ you know?” |
| *Lack of belongingness* | Deductive, RL | Apply when the participant mentions moments in which s/he **did not** feel part of the organization. | “Somehow they push you a little bit out, you know? You are like MSF, but you are a special case. And then it’s, you are a little bit pushed out, I would say. And it’s when you feel less MSF.” |
| *Lack of organizational support* | Inductive, AR | When the participant feels like the organization does not back them up in being able to perform their work. | “They said: ‘Well, as organization, we cannot give you an answer. This means that we cannot provide anything.’” |
| *Legal framework.* | Inductive, AR | MSF operates in many different contexts and countries. Each of these has their own local or regional laws, which create several critical constraints within which the organization needs to find its own maneuvering space. Apply this code to instances when these legally derived elements are mentioned. | “There’s a lot of hatred of us in the world. And the places we go to work are places where that hatred is often rather strong and often legally guardified and international staff joining MSF need to accept that.”  “When I was in [country], not so much (laughs), given what the [of country] law is.” |
| *Living situation* | Deductive, IG | With whom the participant lives, if with anyone. | “Single.” |
| *LGBeneficiaries* | Inductive, OC | When the participant mentions anything concerning sexual minority beneficiaries. | “I’m sure that we have beneficiaries that are lesbian or gay or transsexual, some queer, and whatever.” |
| *LGBNational* | Inductive, OC | When the participant mentions anything concerning sexual minority national staff. | “If a national staff tells you ‘I’m in Uganda, I’m being persecuted as a gay person.’ Knowing as MSF we can also do very little. So, we also have to be realistic in that. So as a kind of support help line, that will be great.” |
| *Married* | Deductive, IG | Whether the participant is married. | “I’m married and live with my partner.” |
| *Mission* | Inductive, AR | Applied when the participant mentions something about MSF’s primary organizational mission, which is “[to] provide assistance to populations in distress, to victims of natural or man-made disasters and to victims of armed conflict, [and to] do so irrespective of race, religion, creed or political convictions.” (See <http://www.msf.org/en/msf-charter-and-principles>). Can also be applied to cases when the participant believes a topic to **not** belong to MSF’s primary goals. | “Well, I mean, the mission I was just on this was in [country], where, there’s been a million refugees from [country] crossing the borders since last July. And, I mean, we had some help posts we had like five help posts doing basic health care. We had a surveillance team that was going around, so that we could have a weekly epidemiology bulletin. But the biggest thing was that there was water scarcity there, and so, the other part of the project was putting in infrastructure for a service water treatment plant, you know, treating two and a half million liters of water a day. So, I mean, you know, I was providing water to, you know, 150.000 people every day. That’s … you know, I mean, those are things that other organizations are just not able to do.” |
| *Motivation* | Deductive, IG | Use this code when the participant mentions their motivation(s) to work for MSF. | “It was about being able to apply [job aspect] in a humanitarian setting.” |
| *Multiple supervisors* | Inductive, OC | Apply when the participant talks about the fact that they’ve had multiple supervisors. | “Soon I will have a new head of department.”  “My previous manager, not my current manager.” |
| *Not married* | Deductive, IG | When the participant has a partner, but is not married. | “I live together with my partner.” |
| *Office* | Deductive, PrE | Apply to any instance in which the participant mentions an aspect connected to an office setting. | “Like I said, in the office I had a talk with someone of HR, who told me during training: ‘Oh, I don’t know whether we have done that before, and I don’t know whether that’s possible.’” |
| *Openness* | Deductive, RL | Apply to instances when the participant discusses the degree to which s/he is open to others within the organization about their sexual orientation. | “I’m open about it. That doesn’t mean that I flaunt with it. But it’s not a secret. My direct colleagues, the ones I have most contact with, they know.” |
| *Organizational culture* | Inductive, AR | This code is used when the participant mentions a specific element of MSF’s organizational culture. | “Because MSF is quite a macho man organization.”  “The way to gain greatest respect or seniority or promotability in MSF, the thing that we value most, is field experience.” |
| *Outed* | Inductive, NR | The moment when the participant’s sexual orientation is disclosed, by someone else, to a third party. | “I got outed to some national staff by a colleague.”  “And she then made a comment, so she apparently knew I had a wife, when there were multiple people around.” |
| *Overdue* | Inductive, annotate data (AD) | Apply to instances when the participant discusses the idea that it has been long enough that MSF has not paid attention to the topic of sexual minority individuals within the organization. | “We can never say it’s too late, yeah, it’s true.”  “It was as if there was, finally, there is just like *some small acknowledgement.*”  “I think that it is long overdue.” |
| *Painful experience* | Inductive, AD | Applied when the participant encountered themselves in a painful situation due to their sexual orientation. Can be shared with the code ‘anecdote.’ | “I have experienced situations where people went on an entire rant against gay people.”  “It’s kind of a shutdown of the conversation in a sense of awkwardness that I’m not used to.” |
| *Patronizing* | Inductive, AR | When the organization is taking responsibility for and deciding about something that might not be completely their responsibility; they might be perceived to be ‘out of line’ by the participant. | “The argument is always, you know, ‘We don’t want to force,’ you know, ‘a particular individual's kind of way of life on national staff or on the culture within the country that we’re working in.’ Which I think is very arrogant, and I think very patronizing as well.” |
| *Personal safety* | Deductive, PrE | Use when personal safety is discussed by the participant. Only apply when the participant discusses their own, individual safety, not when organizational or colleagues’ safety is discussed. | “So the likelihood that something happened to you, as a Western, was very high in that moment.”  “I try to always reason for myself: ‘Can I explain this to my family? That this really is a good idea and that they shouldn’t have to worry.’” |
| *Policy* | Deductive, RL | Apply to any instance in which the participant mentions the presence or absence of an organizational policy. | “I think the fact that the advice from operations is you know, ‘don’t ask don’t tell,’ basically. Particularly when it comes to sexuality. That doesn’t necessarily make it very supportive.” |
| *Principles* | Inductive, AD | Use when the participant mentions the MSF principles. These include Medical ethics, Independence, Impartiality and Neutrality, Bearing witness (Témoignage), and Accountability. Do not use this code when the principle of Témoignage is mentioned; in that case, use code ‘Témoignage.’ | “I thought everybody who joined MSF would have these very high ideals. Around equality, and service provision, and … and all those pieces.”  “And I can identify *my* ideals with that Carta, and that is why I feel a member of MSF and I feel proud of presenting and representing that organization.”  “Because we *do* need to be at the very least a truly neutral and welcoming and respectful organization of everyone.” |
| *Questionnaire* | Inductive, NR | Apply when the participant mentions the questionnaire that was conducted last year by the Rainbow Network, the results of which were presented to the MT and served as a catalyst for their starting up several projects and initiatives. | “We have launched a survey, in which we asked employees, both gay and straight, about their experiences with being LGBQ in the field or their experiences with LGBQ colleagues in the field.” |
| *Recurring* | Inductive, AD | Applied in case something happens repetitively to a participant. | “There’s many times when you don’t necessarily feel comfortable to be out.” |
| *Research* | Inductive, OC | When the participant mentions potential future topics that could be researched within MSF. | “We need another research on that. To see exactly what are these so-called Western values, which MSF maybe is somehow imposing.” |
| *Residence* | Deductive, IG | The place where the participant currently lives. | “Well, [place of residence], yeah.” |
| *Respect* | Inductive, OC | Applied when the participant discusses a basic level of mutual respect for one another. | “You don’t have to have anything in common to be able to have respect for one another.” |
| *Responsibility* | Inductive, AD | When the participant mentions whose responsibility it is, according to them, to, for example, start a debate, lead initiatives, make contributions, etc. | “I think it needs to be collaborative; it can’t just be the Rainbow Network.”  “And then it’s up to the employer to take the torch. And move it forward.” |
| *Role* | Inductive, NR | When the participant mentions their role within the Rainbow Network, and what it is exactly that they contribute to either the Network or the LGBTQ+-community within MSF. | “And so, you know, I’m trying to get more visibility, I mean … energy behind it. But it’s slow-going.”  “So, yeah, I’ve been involved in talking about these initiatives obviously, in the group.” |
| *Sexual orientation* | Deductive, IG | Apply when the participant discusses anything concerning their sexual orientation. | “I have never taken into consideration or thought about my sexual orientation as a constraint to go to any mission.” |
| *Shared experiences* | Inductive, NR | To be applied when the participant mentions that they feel that their experiences as an LGBQ staff member of MSF are shared by others. | “I cannot be the first married lesbian woman in this organization! So there must be other people who already have experience with that.” |
| *Start* | Inductive, NR | When the participant mentions some first steps to be taken by the organization towards a more inclusive work environment for all. Differs from the code ‘initiatives,’ by not indicating specific actions or activities that should be implemented, but rather is a broader code, referring to less concrete actions. | “I think it’s people opening up their eyes, and also allowing room for LGBTQ people to speak. So, creating an opening.”  “I think to start to acknowledge.”  “*If* you join MSF, number one, there’s no tolerance for racism, sexism, homophobia, transphobia or et cetera.” |
| *Strategy* | Inductive, AR | When the participant mentions a specific strategy they apply in their job at MSF. | “You have to have a strategy about when you are going to speak with whom.” |
| *Stress – sexuality-related* | Deductive, RL | Apply when the participant talks about occasions in which s/he feels stress as a result of sexuality-related issues; that is, moments they experience stress due to their sexual orientation. Do not apply when stress is the product of work-related reasons. | “I cannot go through all these kind of painful situations every time that I want to go to a mission.”  “But having been in the field before all this change started happening, it was definitely a stressor. For sure.” |
| *Support – colleague(s)* | Deductive, IG | Use when the participant discusses instances in which s/he felt supported by (one of) their colleague(s). | “It was interesting, because some people *individually*, they sent me mails, supporting me.”  “We received many positive comments, of people saying ‘It’s good that you’re doing this!’” |
| *Support – external* | Deductive, personal experience (PE) | Use when the participant discusses instances in which s/he felt supported by an external party (i.e., friends, family). Only apply to support offered by persons who are not directly linked to MSF. | “And, *many* people were, you know, reached out to me in a positive way, and, you know, continue to stay in contact. When we weren’t in contact before!” |
| *Support – organization* | Deductive, IG | Use when the participant discusses instances in which s/he felt supported by the organization. | “Where, which is very important for me, there’s also room for me as an individual, for example when I’m ill, they don’t make that a big deal.” |
| *Support – supervisor(s)* | Deductive, IG | Use when the participant discusses instances in which s/he felt supported by their supervisor. | “They were generally speaking very supportive as well. It means that it makes kind of a nice feeling, that the people said ‘Okay, it’s great that you’re doing it,’ yeah, ‘if you need time, let us know.’” |
| *Team* | Inductive, NR | When the participant mentions some aspect of their work team. | “You know, it’s clear from the many conversations that I’ve had with, you know, the team members, you know, the national staff that we employ are not perhaps your average citizen in whatever country that we’re working in. You know, they’re highly educated people” |
| *Témoignage* | Inductive, AR | Applied to instances when the participant mentions this idea, which is one of MSF’s guiding principles. Témoignage refers to speaking out, for example about bringing the audience’s attention to something, and taking stance, for example on a certain controversial topic. | “And that means, again, the voice of MSF is often a very important voice. We are heard when we speak. When someone speaks as an MSF person.”  “But I get that the organization doesn’t, or so far didn’t, want to get into the trouble to think about what to do in this case, you know, with this situation, you know… an open discussion about what happened with this.” |
| *Tenure* | Deductive, IG | The period of time that the participant has been working with or for MSF. | “I’m still relatively new to the organization.”  “By now I have a much better view of how things work around here.” |
| *Unclearness* | Inductive, OC | Apply to those parts where the participant mentions something being unclear to them. | “I don’t know if it’s so clear also.”  “You just don’t know what you’re going to be up against.” |
| *Understanding* | Inductive, NR | Apply to those parts of speech when the participant expresses comprehension on something that is, for example, done by MSF or their employees. | “I get it, you know, that our aim is to create awareness towards the management team or for the expats to decide and know what risks they’re taking.” |
| *Valence – negative* | Deductive, PE | Apply to aspects which the participant evaluates in a negative manner. This does not necessarily relate to their sexual orientation. | “But really in the field for me, the most frustrating thing is just when I get teams that won’t work together, or members of teams that are too selfish, narcissistic, and ego-driven.” |
| *Valence – positive* | Deductive, PE | Apply to aspects which the participant evaluates in a positive manner. This does not necessarily relate to their sexual orientation. | “I’m very happy with MSF. Yeah, very fulfilling.”  “I love how dynamic MSF is; I love the energy of it. I love the fact that no two days are the same.” |
| *Visibility* | Inductive, AR | When the participant speaks about the (in)visibility of sexual orientation as a dimension of diversity or about the LGBTQ population within MSF. | “There really aren’t very many of us. There really are not!”  “And also with the goal of increasing visibility of LGBQ staff within MSF.” |
| *Work – current* | Deductive, IG | This code is used when the participant discusses their current occupation, or work activities, positions, and locations within MSF. | “My current work activities are [content of job].” |
| *Work – history* | Deductive, IG | This code is used when the participant discusses their previous occupation, or work activities, positions, and locations within MSF. Do not use if the participant refers to previous work experiences outside of MSF. | “Before this, I was working as a, we call it [job]. It is the person responsible for the [content of job].” |

Codes were added and refined until after having initially coded the third interview, since only a portion of all interviews is used to inductively create codes. Then, memos were taken into consideration, several codes were deleted because they were not widely spread or because they were too specifically describing one particular situation (e.g., *non-action* when the participant described a situation in which no one party undertook action, *oppose* for when the participant mentioned a situation in which s/he actively opposed a decision made by the organization, or *blaming* for when the participant had the idea that they were being blamed for something by the organization), and memos were then put into codes. In total, 52 memos were transformed to codes, which means that, in total, this project made use of 93 codes; 41 of these codes were derived deductively, and 52 of these codes were derived inductively from the data.
